# Urinary peptides in heart failure: a link to molecular pathophysiology

**DOI:** 10.1002/ejhf.2195

**Published:** 2021-05-07

**Authors:** Tianlin He, Michaela Mischak, Andrew L. Clark, Ross T. Campbell, Christian Delles, Javier Díez, Gerasimos Filippatos, Alexandre Mebazaa, John J.V. McMurray, Arantxa González, Julia Raad, Rafael Stroggilos, Helle S. Bosselmann, Archie Campbell, Shona M. Kerr, Colette E. Jackson, Jane A. Cannon, Morten Schou, Nicolas Girerd, Patrick Rossignol, Alex McConnachie, Kasper Rossing, Joost P. Schanstra, Faiez Zannad, Antonia Vlahou, William Mullen, Vera Jankowski, Harald Mischak, Zhenyu Zhang, Jan A. Staessen, Agnieszka Latosinska

**Affiliations:** ^1^ Mosaiques Diagnostics GmbH Hannover Germany; ^2^ Institute for Molecular Cardiovascular Research (IMCAR) RWTH Aachen University Hospital Aachen Germany; ^3^ Academic Cardiology Department Hull York Medical School in the University of Hull Kingston upon Hull UK; ^4^ Institute of Cardiovascular and Medical Sciences University of Glasgow Glasgow UK; ^5^ Program of Cardiovascular Diseases, CIMA Universidad de Navarra IdiSNA and CIBERCV Pamplona Spain; ^6^ Departments of Nephrology and Cardiology Clínica Universidad de Navarra Pamplona Spain; ^7^ Heart Failure Unit, Department of Cardiology Athens University Hospital Attikon Athens Greece; ^8^ Université de Paris, Unité Inserm MASCOT, Department of Anaesthesiology and Intensive Care, Saint Louis‐Lariboisière – Fernand Widal University Hospital, Assistance Publique Hôpitaux de Paris Paris France; ^9^ F‐CRIN INI‐CRCT (Cardiovascular and Renal Clinical Trialists) Nancy France; ^10^ Biotechnology Division, Biomedical Research Foundation Academy of Athens Athens Greece; ^11^ Department of Cardiology, Rigshospitalet University Hospital of Copenhagen Copenhagen Denmark; ^12^ Centre for Genomic and Experimental Medicine, Institute of Genetics & Molecular Medicine University of Edinburgh, Western General Hospital Edinburgh UK; ^13^ MRC Human Genetics Unit, Institute of Genetics & Molecular Medicine University of Edinburgh, Western General Hospital Edinburgh UK; ^14^ Queen Elizabeth University Hospital Glasgow UK; ^15^ Harefield Hospital, Harefield London UK; ^16^ Herlev‐Gentofte Hospital, Department of Cardiology Herlev Denmark; ^17^ Université de Lorraine, Inserm, Centre d'Investigations Cliniques‐ Plurithématique 1433, and Inserm 1116 DCAC, CHRU de Nancy, F‐CRIN INI‐CRCT (Cardiovascular and Renal Clinical Trialists) Nancy France; ^18^ Robertson Centre for Biostatistics, Institute of Health and Wellbeing University of Glasgow Glasgow UK; ^19^ Institut National de la Santé et de la Recherche Médicale, U1048 Institute of Cardiovascular and Metabolic Disease Toulouse France; ^20^ Studies Coordinating Centre, Research Unit Hypertension and Cardiovascular Epidemiology, KU Leuven Department of Cardiovascular Sciences University of Leuven Leuven Belgium; ^21^ Non‐Profit Research Institution Alliance for the Promotion of Preventive Medicine Mechelen Belgium; ^22^ Biomedical Sciences Group, Faculty of Medicine University of Leuven Leuven Belgium

**Keywords:** Biomarker, Collagen, Fibrosis, Heart failure, Proteome, Urine

## Abstract

**Aims:**

Heart failure (HF) is a major public health concern worldwide. The diversity of HF makes it challenging to decipher the underlying complex pathological processes using single biomarkers. We examined the association between urinary peptides and HF with reduced (HFrEF), mid‐range (HFmrEF) and preserved (HFpEF) ejection fraction, defined based on the European Society of Cardiology guidelines, and the links between these peptide biomarkers and molecular pathophysiology.

**Methods and results:**

Analysable data from 5608 participants were available in the Human Urinary Proteome database. The urinary peptide profiles from participants diagnosed with HFrEF, HFmrEF, HFpEF and controls matched for sex, age, estimated glomerular filtration rate, systolic and diastolic blood pressure, diabetes and hypertension were compared applying the Mann–Whitney test, followed by correction for multiple testing. Unsupervised learning algorithms were applied to investigate groups of similar urinary profiles. A total of 577 urinary peptides significantly associated with HF were sequenced, 447 of which (77%) were collagen fragments. *In silico* analysis suggested that urinary biomarker abnormalities in HF principally reflect changes in collagen turnover and immune response, both associated with fibrosis. Unsupervised clustering separated study participants into two clusters, with 83% of non‐HF controls allocated to cluster 1, while 65% of patients with HF were allocated to cluster 2 (*P* < 0.0001). No separation based on HF subtype was detectable.

**Conclusions:**

Heart failure, irrespective of ejection fraction subtype, was associated with differences in abundance of urinary peptides reflecting collagen turnover and inflammation. These peptides should be studied as tools in early detection, prognostication, and prediction of therapeutic response.

## Graphical abstract

Clear differences between urinary peptides from patients with heart failure and controls are observed, indicating alterations in collagen turnover and immune response; while there is a high similarity between urinary profiles among heart failure subtypes. COL1A1, collagen alpha‐1*(I)* chain; COL1A2, collagen alpha‐2(*I*) chain; COL2A1, collagen alpha‐1*(II)* chain; COL3A1, collagen alpha‐1*(III)* chain; FGA, fibrinogen alpha chain; HF, heart failure; HFmrEF, heart failure with mid‐range ejection fraction; HFpEF, heart failure with preserved ejection fraction; HFrEF, heart failure with reduced ejection fraction; IGF, insulin‐like growth factor; UMOD, uromodulin.

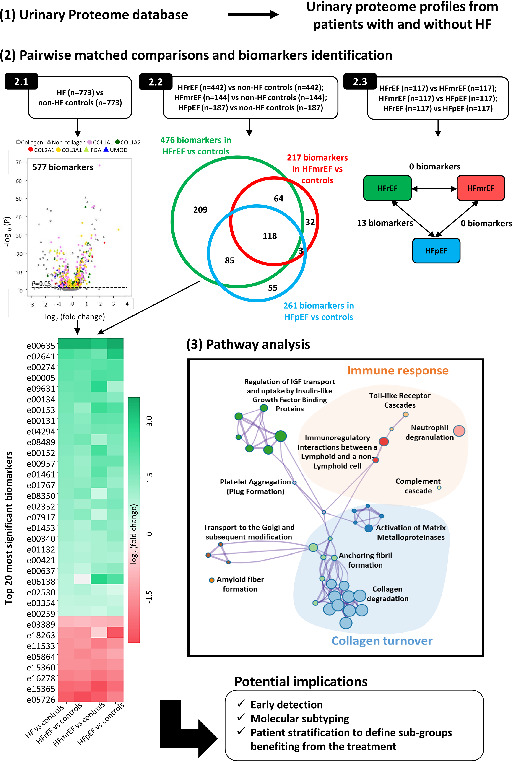

## Introduction

Heart failure (HF) is a syndrome, not a discrete diagnosis, although three subtypes, based on left ventricular ejection fraction (EF), are commonly recognised. However, HF is likely to be far more heterogeneous. Multiple aetiologies (including ischaemic heart disease and hypertension) may lead to abnormalities in the systolic and/or diastolic function of the heart. Because of the similarity in the molecular events underlying many of these clinical pathways, it is challenging to differentiate the molecular processes associated with different subtypes of HF, indicating a need for a deeper understanding of the molecular mechanisms leading to HF. Describing the complex pathophysiology of HF using single biomarkers is challenging and does not reflect the clinical and pathophysiological complexity of the syndrome.

The application of proteome analysis to clinically relevant problems is an emerging and promising field of biomarker research.^1^ Previous studies using urinary proteome analysis^2^ have identified urinary peptides strongly associated with renal damage, coronary artery disease and left ventricular diastolic dysfunction.

The aim of the present study was a comprehensive description of molecular differences at the level of the urinary proteome between individuals with HF and without HF, as well as between HF subtypes. The focus was placed on peptides with high specificity for HF based on the comparison between case and control groups matched for kidney function, sex, age, blood pressure and the presence of diabetes and hypertension.

## Methods

### Patient data

We extracted patient data from the Human Urinary Proteome database of capillary electrophoresis mass spectrometry (CE‐MS) data on urinary peptides^3^, ^4^ that at the time of the extraction contained datasets from 50 651 individuals. Data were included in the study if: (i) data on sex, age, estimated glomerular filtration rate (eGFR), systolic (SBP) and diastolic blood pressure (DBP), and diabetic status were available; (ii) age was above 20 years; (iii) data passed quality control criteria for CE‐MS measurements, post‐acquisition data processing and calibration, as previously described.^4^ Exclusion criteria were diagnosis of cancer, solid organ transplantation, or intensive care unit stay at the time of inclusion. Urine samples from 5608 participants (individuals with and without HF) were available. Overall, 1180 patients had HF and 4428 patients did not have a diagnosis of HF.

Urine samples were obtained from the following studies: (i) STANISLAS cohort study^5^; (ii) Hull LifeLab^6^; (iii) study on diagnostic and prognostic utility of the proteomic classifier HF1^7^; (iv) FLEMENGHO and EPOGH^8^; (v) Generation Scotland cohort^9^; (vi) an echocardiographic and cardiac biomarker study^10^; and (vii) cross‐sectional multicentre cohort study.^11^ Further details such as patients' characteristics and information on EF measurements, if applicable, are reported in the relevant manuscripts. For 82% of HF patients, urine samples were collected within 24 h of the assessment of the EF. For remaining HF patients, timing was not recorded. The investigation conforms with the principles outlines in the Declaration of Helsinki^12^; all data were anonymized and the study was approved by the ethics committee of the University Aachen (EK163/19).

### Data curation

Heart failure was defined using the European Society of Cardiology (ESC) guidelines recommended at the time of patients' recruitment. In all cases, the diagnosis was based on the presence of symptoms or signs of HF, caused by a structural and/or functional heart abnormality. Patients with HF with missing measurements of EF or natriuretic peptides [N‐terminal prohormone of B‐type natriuretic peptide (NT‐proBNP) or B‐type natriuretic peptide (BNP)] were excluded (*n* = 154). Only patients with NT‐proBNP or BNP >125 pg/mL or >35 pg/mL were included. To account for an update in the ESC guidelines, the diagnosis of HF subtypes was assigned based on the EF thresholds in the ESC recommendations^13^ [i.e. for HF with reduced EF (HFrEF): EF <40%; for HF with mid‐range EF (HFmrEF): EF ≥40 and ≤49; for HF with preserved EF (HFpEF): EF ≥50%]. Based on these criteria, 515, 259 and 219 subjects with the diagnosis of HFrEF, HFmrEF and HFpEF were identified, respectively.

**Figure 1:**
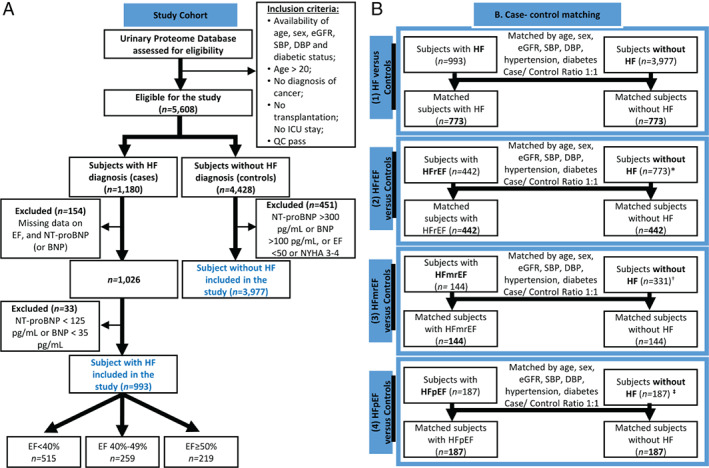
Study design. Consort diagram for patient selection (*A*) as well as case‐control matching workflow (*B*) are presented. BNP, B‐type natriuretic peptide; DBP, diastolic blood pressure; EF, ejection fraction; eGFR, estimated glomerular filtration rate; HF, heart failure; HFmrEF, heart failure with mid‐range ejection fraction; HFpEF, heart failure with preserved ejection fraction; HFrEF, heart failure with reduced ejection fraction; ICU, intensive care unit; NT‐proBNP, N‐terminal prohormone of pro‐B‐type natriuretic peptide; QC, quality control; SBP, systolic blood pressure. *When performing case‐control matching for each HF subtype, only controls (*n* = 773) that were matched in the comparison of all patients with HF (*n* = 773) were considered. ^†^When matching controls for patients with HFmrEF (*n* = 144), controls that have been matched to HFrEF (*n* = 442) were not considered. ^**‡**^When matching controls to patients with HFpEF (*n* = 187), controls that were matched in previous comparisons were excluded.

Individuals without HF with NT‐proBNP >300 pg/mL (or BNP >100 pg/mL), EF <50% or with New York Heart Association (NYHA) III–IV symptoms were excluded from the control group (*n* = 451). The non‐HF control group consisted of individuals who at the time of urine sampling had no known diagnosis of HF (*n* = 3977). The latter group included, among others, healthy individuals, patients with chronic kidney disease (CKD), hypertension, diabetes or coronary artery disease (or a combination of these pathologies). CKD was defined based on the clinical diagnosis and/or on the kidney function (eGFR < 60 mL/min/1.73 m^2^). eGFR was estimated based on the Chronic Kidney Disease Epidemiology Collaboration (CKD‐EPI) equation. Hypertension was diagnosed based on the use of antihypertensive drugs, SBP of 140 mmHg or higher, and/or DBP of 90 mmHg or higher.^14^ Patient selection and data curation are graphically depicted in *Figure* *1A*.

### Case‐control matching

Matching of cases to non‐HF controls was performed in R (‘MatchIt’^15^). A ‘nearest neighbour’ matching‐method was applied, allowing for discarding of datasets from both groups that were outside the distance‐measure and re‐estimation of distance‐measure after discarding. Logistic regression was used to estimate the distance‐measure (default setting). Case‐control matching at a 1:1 ratio was performed based on sex, age, eGFR, SBP, DBP, and the presence of diabetes and hypertension. HF patients were matched to non‐HF controls. Those patients were divided based on EF, followed by matching of the controls selected from the group of controls already matched to the complete HF cohort. Patients presenting with specific HF subtype were subsequently matched to each other. The following groups were matched based on these variables: (i) HF vs. non‐HF controls, (ii) HFrEF vs. non‐HF controls, (iii) HFmrEF vs. non‐HF controls, (iv) HFpEF vs. non‐HF controls, (v) HFrEF vs. HFmrEF, (vi) HFmrEF vs. HFpEF, and (vii) HFrEF vs. HFpEF. The schematic illustration of the matching is presented in *Figure* *1B* and online supplementary *Figure* *S1*.

### Capillary electrophoresis mass spectrometry

Preparation and measurements of urine samples using CE‐MS were conducted as described previously.^4^ CE‐MS analysis was performed with a P/ACE MDQ CE (Beckman Coulter, Fullerton, CA, USA) coupled to a micro‐TOF‐MS (Bruker Daltonic, Bremen, Germany). Raw MS data were evaluated using MosaFinder^3^ applying a probabilistic clustering algorithm and using both isotopic distributions and conjugated masses for charge state determination. Normalization of the CE‐MS data was based on 29 collagen fragments that are generally not affected by disease and serve as internal standards.^3^, ^4^ Missing values were interpreted as zero. Identified HF biomarkers were assigned *in silico* to sequenced peptides from the Human Urinary Proteome database as described elsewhere.^3^ Further information on CE‐MS analysis and peptide sequencing are provided in online supplementary *Methods*.

### Statistical analysis

Statistical analysis of urinary proteome profiles was performed using the non‐parametric Mann–Whitney test.^16^ A Benjamini–Hochberg (BH) approach was employed to correct for multiple testing and the false discovery rate. An adjusted *P*‐value < 0.05 was considered statistically significant. Only peptides that were found at >30% frequency in cases or controls were examined, following the standard procedure for defining disease‐associated peptides. Correlation analysis was performed using Spearman's rank‐order correlation.

### Unsupervised classification

Unsupervised clustering was performed using the R package ‘ConsensusClusterPlus’.^17^ Abundances for sequenced peptides (independent of the significance level) were transformed to ranks, and pairwise correlations between samples were calculated with Spearman's rank correlation coefficient. The correlation matrix was submitted for agglomerative hierarchical clustering and using clustering centroids and average linkage^17^ for a pre‐defined number of solutions (tested 2–8 clusters). Class assignment for each solution was determined after 1000 permutations by resampling 95% of the sample set, and the output was evaluated based on the consensus heat maps, and cluster size.^17^ Principal component analysis (PCA) was conducted on the same input data, using the base R function ‘prcomp()’ and the argument scale = FALSE.

### Bioinformatic analysis

Proteases responsible for the generation of the urinary peptide fragments were predicted using the Proteasix tool.^18^ Predicted proteases and proteins corresponding to sequenced urinary peptides were included as an input for functional analysis. Functional analysis was performed with the Metascape,^19^ with Reactome pathway database used as an ontology source (online supplementary *Methods*).

## Results

### Cohort characteristics

Among the 4970 participants included in the study after data curation (*Figure* *1A*), case‐control matching resulted in the selection of 773 patients with HF and 773 controls. Cases were stratified into three subtypes: HFrEF (*n* = 442), HFmrEF (*n* = 144) and HFpEF (*n* = 187). For each of the subtypes, the same number of controls were matched (*Figure* *1B*). The key matching characteristics were similar between groups (Mann–Whitney test, *P* > 0.05), as shown in *Table* *1*. Patients with HFrEF, HFmrEF and HFpEF were matched to each other, resulting in the selection of 117 individuals in each group. The characteristics of the matched HF patients are presented in online supplementary *Table* *S1*.

**Table 1:** Characteristics of the matched patients

Characteristics	HF vs. Controls			HFrEF vs. Controls			HFmrEF vs. Controls			HFpEF vs. Controls		
	HF (*n* = 773)	Controls (*n* = 773)	*P*‐value	HFrEF (*n* = 442)	Controls (*n* = 442)	*P*‐value	HFmrEF (*n* = 144)	Controls (*n* = 144)	*P*‐value	HFpEF (*n* = 187)	Controls (*n* = 187)	*P*‐value
Age (years)	71.00 [64.00–77.00]	71.00 [65.00–76.00]	0.8956	71.00 [64.00–77.00]	72.00 [64.00–77.00]	0.9184	68.00 [62.00–72.00]	68.00 [64.00–72.00]	0.8136	73.00 [68.00–78.00]	73.00 [68.00–76.75]	0.7908
Female sex, *n* (%)	257 (33.2)	257 (33.2)	0.9569	128 (29.0)	126 (28.5)	0.9408	43 (29.9)	47 (32.6)	0.7029	86 (46.0)	84 (44.9)	0.9173
SBP (mmHg)	133.00 [120.00–150.00]	136.00 [123.00–148.00]	0.0637	131.00 [116.00–144.00]	132.00 [121.00–146.00]	0.0565	137.00 [126.00–153.00]	138.50 [127.00–147.00]	0.2869	135.00 [121.25–154.00]	139.0000 [127.25–151.75]	0.0632
DBP (mmHg)	74.00 [66.00–82.00]	75.00 [69.00–81.00]	0.1357	73.00 [65.00–82.00]	75.00 [69.00–81.00]	0.1562	78.00 [70.00–84.50]	76.00 [70.00–83.50]	0.6028	70.00 [64.00–82.00]	75.00 [68.00–80.00]	0.1332
Heart rate (bpm)	71.00 [60.00–82.00]	65.00 [58.00–73.00]	***<* 0.0001**	71.00 [60.00–82.00]	64.00 [59.00–72.50]	**< 0.0001**	71.00 [61.00–80.00]	66.00 [58.00–75.00]	**0.0158**	70.00 [60.00–80.00]	65.00 [58.00–73.00]	**0.0014**
Body mass index (kg/m^2^)	28.54 [24.85–32.96]	27.10 [24.56–29.89]	***<* 0.0001**	27.60 [23.85–31.24]	27.25 [24.93–29.87]	0.7036	29.31 [25.74–35.85]	26.88 [24.55–29.47]	**0.0001**	31.01 [27.05–35.78]	26.64 [23.91–30.54]	***<* 0.0001**
eGFR^a^ (mL/min/1.73 m^2^)	64.02 [47.68–81.33]	67.23 [47.95–83.92]	0.3170	63.70 [45.83–80.47]	66.17 [42.46–82.27]	0.9964	69.17 [54.40–88.77]	75.77 [59.50–89.94]	0.1570	61.79 [47.87–77.55]	65.72 [46.39–80.42]	0.4979
**Clinical features of HF**												
EF (%)	38.00 [30.00–48.00]	68.00 [62.00–72.77]	***<* 0.0001**	31.00 [25.00–35.00]	68.00 [62.00–72.00]	***<* 0.0001**	43.00 [41.00–46.00]	68.00 [62.35–73.98]	***<* 0.0001**	56.00 [53.00–62.00]	68.00 [64.00–73.00]	***<* 0.0001**
NT‐proBNP (pg/mL)	806.50 [389.00–1785.50]	144.91 [75.45–211.89]	***<* 0.0001**	1140.55 [479.00–2496.75]	141.70 [68.13–203.95]	**< 0.0001**	478.20 [276.75–860.25]	132.72 [75.81–216.36]	***<* 0.0001**	692.00 [330.00–1166.75]	170.00 [115.00–221.00]	***<* 0.0001**
BNP (pg/mL)	397.00 [186.75–819.25]	31.00 [19.00–47.00]	***<* 0.0001**	505.50 [230.00–1052.00]	28.50 [18.65–48.00]	***<* 0.0001**	212.00 [116.00–426.25]	24.00 [13.50–49.45]	***<* 0.0001**	336.00 [154.00–524.50]	33.50 [24.75–47.25]	***<* 0.0001**
NYHA functional class, *n* (%)			***<* 0.0001**			***<* 0.0001**			***<* 0.0001**			***<* 0.0001**
0	6 (0.8)	505 (65.3)		5 (1.1)	283 (64.0)		1 (0.7)	107 (74.3)		0 (0.0)	115 (61.5)	
I	128 (16.6)	67 (8.7)		57 (12.9)	37 (8.4)		30 (20.8)	12 (8.3)		41 (21.9)	18 (9.6)	
II	420 (54.3)	4 (0.5)		220 (49.8)	4 (0.9)		95 (66.0)	0 (0.0)		105 (56.1)	0 (0.0)	
III	197 (25.5)	0 (0.0)		143 (32.4)	0 (0.0)		17 (11.8)	0 (0.0)		37 (19.8)	0 (0.0)	
IV	22 (2.8)	0 (0.0)		17 (3.9)	0 (0.0)		1 (0.7)	0 (0.0)		4 (2.1)	0 (0.0)	
Unknown	0 (0.0)	197 (25.5)		0 (0.0)	118 (26.7)		0 (0.0)	25 (17.4)		0 (0.0)	54 (28.9)	
**Medical history, *n* (%)**												
Hypertension	507 (65.6)	508 (65.7)	1.0000	294 (66.5)	295 (66.7)	1.0000	100 (69.4)	95 (66.0)	0.6142	113 (60.4)	118 (63.1)	0.6704
Diabetes	263 (34.0)	253 (32.7)	0.6274	138 (31.2)	133 (30.1)	0.7704	48 (33.3)	48 (33.3)	0.9005	77 (41.2)	72 (38.5)	0.6727
CAD	382 (49.4)	78 (10.1)	***<* 0.0001**	238 (53.8)	42 (9.5)	***<* 0.0001**	66 (45.8)	13 (9.0)	**0.0001**	78 (41.7)	23 (12.3)	***<* 0.0001**
CKD^b^	339 (43.9)	335 (43.3)	0.8777	198 (44.8)	197 (44.6)	1.0000	54 (37.5)	54 (37.5)	0.9031	87 (46.5)	84 (44.9)	0.8355

Values are presented as median [interquartile range] or *n* (%).

Mann–Whitney test was used for continuous variables, while Chi‐squared test was applied for categorical variables. Bold indicates *P* < 0.05.

BNP, brain natriuretic peptide; CAD, coronary artery disease; CKD, chronic kidney disease; DBP, diastolic blood pressure; EF, ejection fraction; eGFR, estimated glomerular filtration rate; HF, heart failure; HFmrEF, heart failure with mid‐range ejection fraction; HFpEF, heart failure with preserved ejection fraction; HFrEF, heart failure with reduced ejection fraction; NT‐proBNP, N‐terminal prohormone of B‐type natriuretic peptide; NYHA, New York Heart Association; SBP, systolic blood pressure.

^a^ eGFR was estimated based on the Chronic Kidney Disease Epidemiology Collaboration (CKD‐EPI) equation.

^**b**^ CKD was defined based on clinical diagnosis and/or kidney function (eGFR < 60 mL/min/1.73 m^2^).

### Urinary peptide differences in heart failure

**Figure 2:**
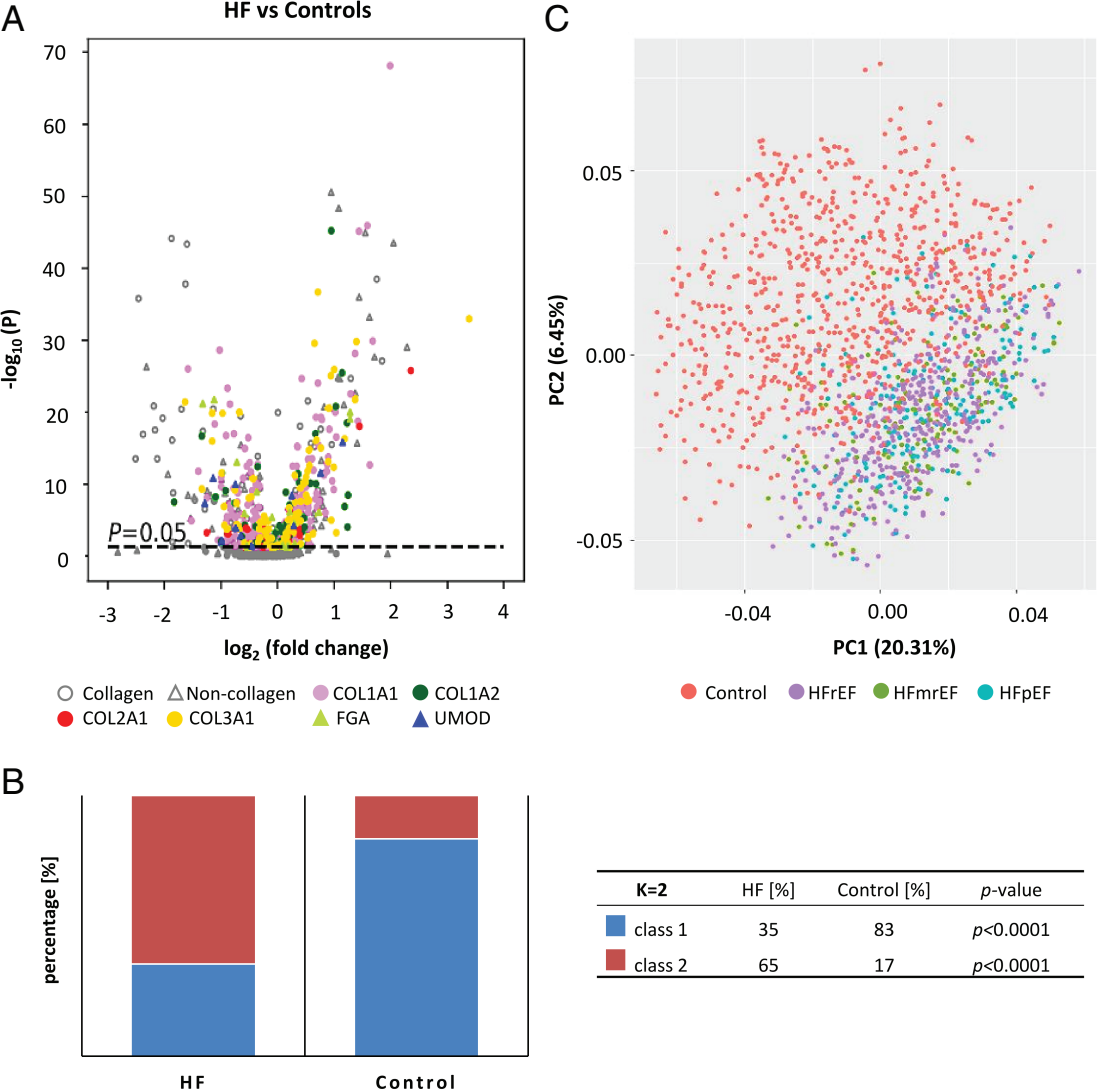
Urinary peptides in heart failure (HF). (*A*) Volcano plot showing sequenced peptides identified between patients with HF and matched controls. Directionality of the difference, magnitude as well as significance level [Benjamini–Hochberg (BH) adjusted *P*‐value] are displayed. Discrimination between collagen and non‐collagen‐derived peptides is provided. Peptides originated from proteins for which at least 10 significant peptides were identified (*P* < 0.05, BH adjusted) when comparing all patients with HF and controls are colour‐coded. (*B*) Segregation of all study participants into two classes based on consensus clustering. (*C*) Graphical representation of the separation of the study participants using principal component analysis. Principal component analysis was performed based on sequenced peptides detected in 30% of samples (independent of the significance level), for which abundances were transformed to ranks. COL1A1, collagen alpha‐1*(I)* chain; COL1A2, collagen alpha‐2(*I*) chain; COL2A1, collagen alpha‐1*(II)* chain; COL3A1, collagen alpha‐1*(III)* chain; FGA, fibrinogen alpha chain; HFmrEF, heart failure with mid‐range ejection fraction; HFpEF, heart failure with preserved ejection fraction; HFrEF, heart failure with reduced ejection fraction; UMOD, uromodulin.

In this study, 3332 sequenced urinary peptides were investigated. A total of 577 peptides differed significantly (BH adjustment *P* < 0.05) between patients with HF and controls. The top 20 peptides with the lowest *P*‐value for differential expression between groups are listed in *Table* *2*, while their frequency, average abundance and discrimination metrics [e.g. area under the receiver operating characteristic curve (AUC), sensitivity, specificity] for distinguishing between patients with HF and controls are provided in online supplementary *Table* *S2*. AUCs were in a range between 0.65 and 0.75, with a median value of 0.7. Most of the peptides associated with HF compared with controls were collagen fragments [447 of 577 peptides (77%)] (*Figure* *2A*), with collagen alpha‐1(I) chain, collagen alpha‐1(III) chain and collagen alpha‐2(I) chain represented by the largest number of peptide fragments.

**Table 2:** List of the 20 peptides providing the greatest discrimination between patients with heart failure (HF, *n* = 773) and matched controls (*n* = 773) as well as between matched controls and patients with HF with reduced (HFrEF, *n* = 442), mid‐range (HFmrEF, *n* = 144) and preserved ejection fraction (HFpEF, *n* = 187)

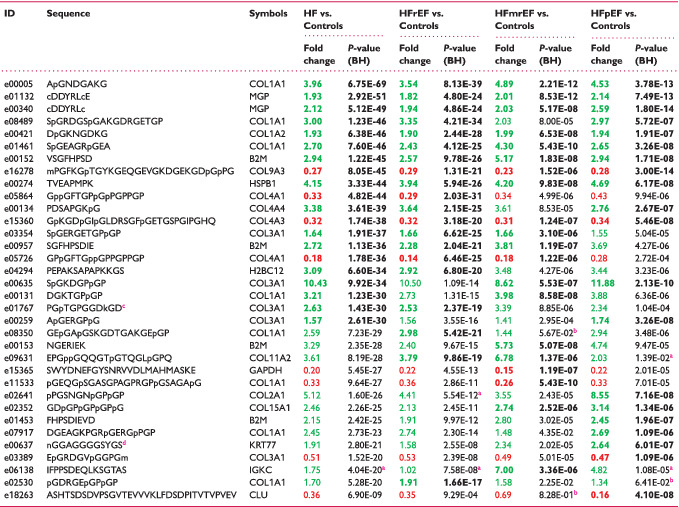

BH, Benjamini–Hochberg.

Peptides are ordered by increasing *P*‐value in HF vs. controls.

Bold indicates top 20 peptides in individual comparisons.

Peptides higher in disease are labelled in green, lower in red.

^a^ Peptides that did not pass the frequency threshold of 30%.

^b^ Peptides that did not pass the *P*‐value (BH adjusted) threshold of 0.05.

^c^ Posttranslational modification: formylation (K).

^d^ Posttranslational modification: deamination (N).

The results were consistent when investigating significant peptides in HF patients stratified based on the original cohort (online supplementary *Table* *S3* and *Figure* *S2*), no batch effect was detectable. Along these lines, our findings were also consistent across patients with acute and chronic HF (online supplementary *Table* *S4* and *Figure* *S3*).

To assess the specificity of the findings for HF, we compared the 577 peptides that were significantly different between patients with HF and controls with the 273 peptides significantly associated with CKD in a previous study (CKD273^20^). The biomarkers were defined in two independent cohorts. Overlapping biomarkers (71 peptides) as well as biomarkers unique for HF (506 peptides) and CKD (202 peptides) were observed (online supplementary *Figure* *S4*). The directional change in 32 of these 71 peptides was opposite in HF and CKD, and only very weak correlation between the fold‐change in the peptide abundances observed in CKD and HF was detectable (Spearman's coefficient of rank correlation (rho) = 0.25, *P* = 0.03, *n* = 71). Moreover, we investigated an association of peptide abundance with eGFR (used as a continuous variable) in patients with HF. Seventy‐three peptides showed significant correlation (*P* < 0.05 BH adjusted), with rho value above 0.2. An additional 210 peptides *(P* < 0.05 BH adjusted) had rho values below 0.2, indicating very weak/negligible relationship.

Using consensus clustering, the study participants were separated into two clusters. The majority of patients with HF (65%) were allocated to cluster 2, while 83% of non‐HF controls were allocated to cluster 1 (Chi‐squared test, *P* < 0.0001) (*Figure* *2B*). There was no separation based on HF subtype (Chi‐squared test, *P* > 0.05) (online supplementary *Figure* *S5*). Findings applying PCA were similar, separating HF from controls, but no evidence for discrimination between HF subtypes (*Figure* *2C*).

### Urinary peptide differences in heart failure subtypes

There were only minor (or no) differences between the three HF subtypes in pairwise comparisons. Only 13 peptides were significantly different between HFrEF and HFpEF **(**online supplementary *Table* *S5*), and none were detected with a *P*‐value < 10^−5^. AUCs were lower when comparing to those peptides differentiating between patients with HF and controls, and were in a range between 0.61 and 0.67, with a median value of 0.63.

Combining the urinary peptides with best discriminatory ability (based on the AUC) into classifiers using support vector machines did not enable reliable differentiation of the subtypes of HF (online supplementary *Figure* *S6*), consistently with the results from unsupervised clustering and PCA.

**Figure 3:**
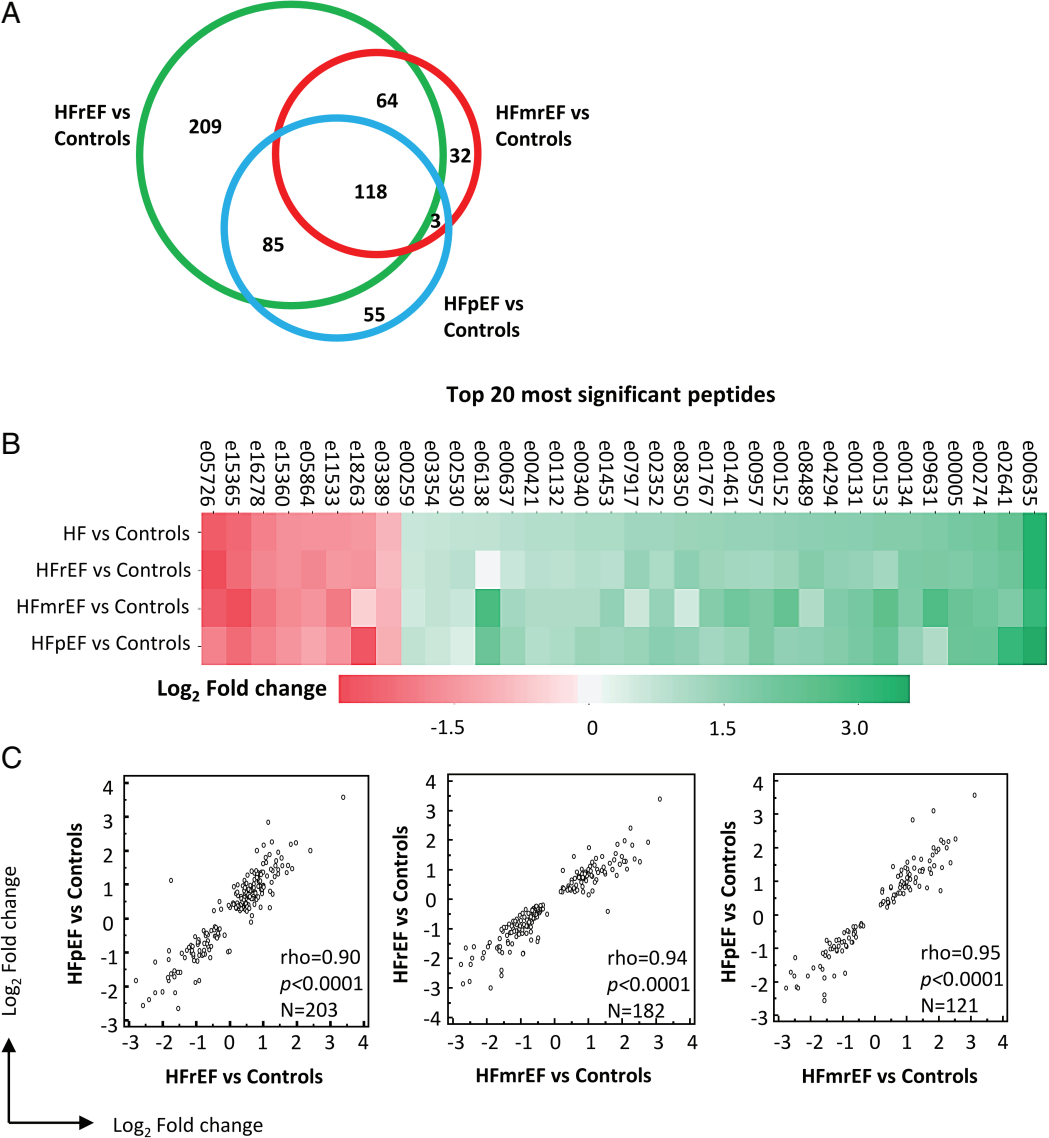
Urinary peptides across heart failure (HF) subtypes. (*A*) Comparison of the sequenced peptides that significantly differ between analysed sub‐groups. (*B*) Heat map presenting log2 transformed fold‐changes for 20 most significant peptides across HF subtypes and between all patients with HF in comparison to controls (*Table* *2*). (*C*) Correlation of peptides significantly associated with HF with preserved (HFpEF), reduced (HFrEF) and mid‐range (HFmrEF) ejection fraction when comparing the fold‐change observed in HFpEF vs. controls, HFrEF vs. controls and HFmrEF vs. controls. Commonly significant peptides between comparisons were included.

We further investigated differences in urinary peptides between patients with HFrEF, HFmrEF and HFpEF and non‐HF controls. In matched comparisons, 476 peptides were significantly different between patients with HFrEF and controls, 217 between patients with HFmrEF and controls, and 261 between patients with HFpEF and controls (*Figure* *3A*). For many of these peptides, the differences in abundance were detected with *P*‐values < 10^−5^ (online supplementary *Figure* *S7*). The peptides that were most significantly different between groups are listed in *Table* *2*, while further details of frequency, average abundance and discrimination metrics are itemised in online supplementary *Table* *S2*. AUCs were in a range between 0.64 and 0.77, with a median value of 0.69. A very high degree of consistency among the biomarkers differentiating individual HF subtypes from controls appears evident (*Figure* *3B*). A strong correlation between the fold changes in peptide abundance when comparing control subjects to patients with HFrEF or HFpEF was observed. Similar results were obtained for patients with HFmrEF (*Figure* *3C*).

### Bioinformatic analysis

**Figure 4:**
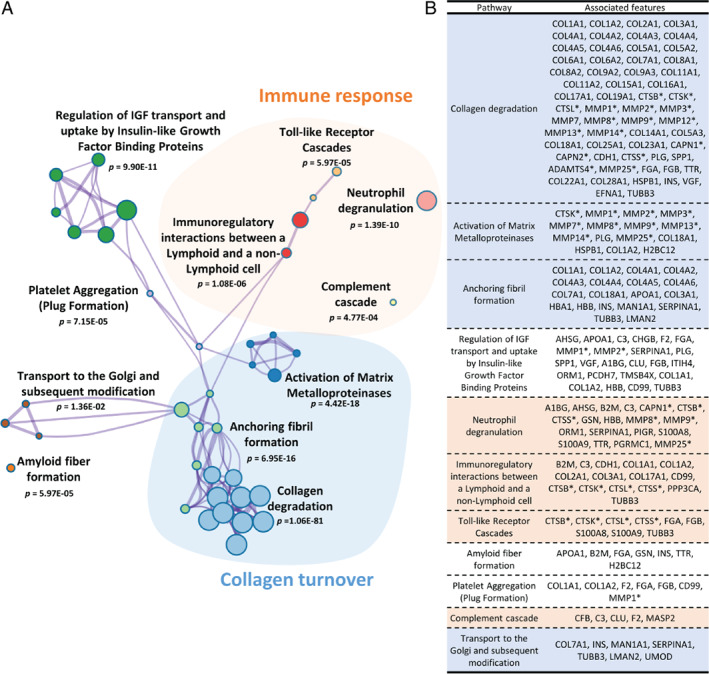
Enrichment analysis based on Reactome pathways. (*A*) Network of enriched terms. Graphical representation of pathways significantly enriched based on the predicted proteases and proteins representing the urinary peptides. Networks are coloured based on cluster ID, the thickness of the edge represents the similarity score. The most significant term from each cluster was selected as label and for those terms, *P*‐value corrected using Banjamini–Hochberg (BH) procedure is given. Among the most prominent findings, significant enrichment for pathways related to collagen turnover (highlighted in blue) and immune response (highlighted in orange) was observed. (*B*) Parental proteins and predicted proteases annotated to the most significant terms. A1BG, alpha‐1B‐glycoprotein; ADAMTS4, A disintegrin and metalloproteinase with thrombospondin motifs 4; AHSG, alpha‐2‐HS‐glycoprotein; APOA1, apolipoprotein A‐I; B2M, beta‐2‐microglobulin form pI 5.3; C3, complement C3; CAPN1, calpain‐1 catalytic subunit; CAPN2, calpain‐2 catalytic subunit; CD99, CD99 antigen; CDH1, cadherin 1; CFB, complement factor B; CHGB, secretogranin‐1; CLU, clusterin; COL11A1, collagen alpha‐1(XI) chain; COL11A2, collagen alpha‐2(XI) chain; COL14A1, collagen alpha‐1(XIV) chain; COL15A1, collagen alpha‐1(XV) chain; COL16A1, collagen alpha‐1(XVI) chain; COL17A1, collagen alpha‐1(XVII) chain; COL18A1, collagen alpha‐1(XVIII) chain; COL19A1, collagen alpha‐1(XIX) chain; COL1A1, collagen alpha‐1(I) chain; COL1A2, collagen alpha‐2(I) chain; COL22A1, collagen alpha‐1(XXII) chain; COL23A1, collagen alpha‐1(XXIII) chain; COL25A1, collagen alpha‐1(XXV) chain; COL28A1, collagen alpha‐1(XXVIII) chain; COL2A1, collagen alpha‐1(II) chain; COL3A1, collagen alpha‐1(III) chain; COL4A1, collagen alpha‐1(IV) chain; COL4A2, collagen alpha‐2(IV) chain; COL4A3, collagen alpha‐3(IV) chain; COL4A4, collagen alpha‐4(IV) chain; COL4A5, collagen alpha‐5(IV) chain; COL4A6, collagen alpha‐6(IV) chain; COL5A1, collagen alpha‐1(V) chain; COL5A2, collagen alpha‐2(V) chain; COL5A3, collagen alpha‐3(V) chain; COL6A1, collagen alpha‐1(VI) chain; COL6A2, collagen alpha‐2(VI) chain; COL7A1, collagen alpha‐1(VII) chain; COL8A1, collagen alpha‐1(VIII) chain; COL8A2, collagen alpha‐2(VIII) chain; COL9A2, collagen alpha‐2(IX) chain; COL9A3, collagen alpha‐3(IX) chain; CTSB, cathepsin B; CTSK, cathepsin K; CTSL, cathepsin L1; CTSS, cathepsin S; EFNA1, ephrin‐A1; F2, thrombin light chain; FGA, fibrinogen alpha chain; FGB, fibrinogen beta chain; GSN, gelsolin; H2BC12, histone H2B type 1‐K; HBA1, haemoglobin subunit alpha; HBB, haemoglobin subunit beta; HSPB1, heat shock protein beta‐1; INS, insulin; ITIH4, 35 kDa inter‐alpha‐trypsin inhibitor heavy chain H4; LMAN2, vesicular integral‐membrane protein VIP36; MAN1A1, mannosyl‐oligosaccharide 1;2‐alpha‐mannosidase IA; MASP2, mannan‐binding lectin serine protease 2; MMP1, interstitial collagenase; MMP12, macrophage metalloelastase; MMP13, collagenase 3; MMP14, matrix metalloproteinase‐14; MMP2, 72 kDa type IV collagenase; MMP25, matrix metalloproteinase‐25; MMP3, stromelysin‐1; MMP7, matrilysin; MMP8, neutrophil collagenase; MMP9, matrix metalloproteinase‐9; ORM1, alpha‐1‐acid glycoprotein 1; PCDH7, protocadherin‐7; PGRMC1, membrane‐associated progesterone receptor component 1; PIGR, polymeric immunoglobulin receptor; PLG, plasminogen; PPP3CA, serine/threonine‐protein phosphatase; S100A8, protein S100‐A8; S100A9, protein S100‐A9; SERPINA1, alpha‐1‐antitrypsin; SPP1, osteopontin; TMSB4X, thymosin beta‐4; TTR, transthyretin; TUBB3, tubulin beta‐3 chain; UMOD, uromodulin; VGF, neurosecretory protein VGF. *In silico predicted proteases.

*In silico* protease analysis based on the peptides differing between HF and controls resulted in the identification of 76 proteases. Eighteen proteases that had at least one protease/cleavage site association reported in the literature and the percentage of cleavage events above 1% (the most active) were shortlisted (online supplementary *Table* *S6*). Shortlisted proteases included matrix metalloproteinases, calpains, cathepsins, and a disintegrin and metalloproteinase with thrombospondin motifs. Pathway enrichment analysis based on the shortlisted predicted proteases, along with proteins representing the peptides significantly different between patients with HF and controls, was conducted. A network of significantly enriched terms is presented in *Figure* *4*. The analyses also indicated a high degree of similarity in predicted proteases and enriched pathways between the three HF subtypes.

## Discussion

This is the first investigation of the urinary proteomic profile of more than 1500 patients with HF encompassing the full spectrum of EF and non‐HF controls (*Graphical Abstract*). While our data demonstrate highly significant differences between patients with HF and non‐HF controls for multiple urinary peptides, there were very few differences among the different HF subtypes defined by EF. Our study was also designed to identify HF specific peptides. To limit the potential impact of co‐existing diseases (CKD) and various other factors (e.g. age) on urinary peptides, analysis was performed in matched cohorts. Identified peptides significantly associated with HF were clearly distinct from those peptides associated with CKD, supporting their specificity for HF.

Our data demonstrate highly significant differences in multiple urinary peptides between patients with HF and matched controls. A frequency threshold of 30% was applied when defining peptides significantly associated with HF. This threshold has been found optimal in previous studies where multiple peptides were combined into a classifier. Although biomarkers with low frequency are of limited value on their own, they are beneficial in biomarker panels since a higher number of biomarkers in the panel improves stability.^4^, ^16^ The most prominent alterations identified appear to reflect dysregulation of collagen turnover, a well‐known biological process in HF,^21^ with specific collagen peptides affected in HF. An explanation for this observation may be alterations in the activity of proteases, which consequently would result in variation in the abundance of peptides representing corresponding pairs of substrates and products. These findings contrast to CKD, where collagen‐related peptides were consistently reduced.^20^

While we do not have a definitive explanation for the increases and decreases of specific peptides and the exact mechanism behind this observation is yet unknown, our findings are highly consistent across individual comparisons. We found a general reduction in fragments derived from network‐forming collagens (collagen type IV, and VIII) in patients with HF, suggesting that disturbance in collagen degradation, possibly due to increasing stability of collagen networks and cross‐linking that protects them from degradation. Our findings are consistent with the observation that all major HF subtypes are associated with cardiac fibrosis and potentially also fibrosis of other organs and tissues including arteries, where collagen degradation may play a major role.

Circulating biomarkers reflecting collagen metabolism have been shown to be associated with histologically proven myocardial fibrosis in HF (carboxy‐terminal propeptide of procollagen type I, amino‐terminal propeptide of procollagen type III, and collagen type I telopeptide‐to‐serum matrix metalloproteinase‐1 ratio).^21^ However, these peptides are not specific for cardiac fibrosis and may indicate abnormalities of collagen turnover in other organs.^22^ Urinary peptides also may reflect molecular changes induced by systemic conditions or localised diseases. While we cannot confirm the origin of the specific urinary peptides in this study, the fact that they differ largely from those identified in patients with CKD suggests that the changes observed are specific for HF. Although this was an historical comparison, biomarkers associated with CKD^20^ and HF (as defined in this study) were identified using the same technological platform. The 273 biomarkers for CKD have been consistently used over more than 10 years. Therefore, the limited overlap between CKD and HF biomarkers cannot be due to changes in biomarker definition over time. In addition, there was a very weak association of the abundance of HF‐associated peptides with eGFR in patients with HF, which further supports their specificity for HF. It is well‐recognised that HF and CKD commonly co‐exist, share many common risk factors, and are expected to share common mechanisms.^23^ However, our study was designed to find peptides that are specific for HF (and not other comorbidities like CKD). To eliminate the impact of CKD (and other variables) on urinary peptides, the urinary peptide profiles from participants with HF and controls were carefully matched to, among others, obtain an even distribution of CKD (based on eGFR). As a result, we expect changes specific for CKD to be eliminated. This may partially explain the low overlap between HF and CKD‐associated peptides, since impact of CKD was removed through patients matching. Investigation of overlapping urinary peptides in HF patients with CKD and without CKD was beyond the scope of this work and will be examined in a future study. Urinary proteome analysis appears to be biased towards identification of collagen fragments.^2^ Collagen peptides are enriched in urine, possibly because they are not reabsorbed in the renal tubules.^2^ However, in addition to changes in specific collagen fragments, we detected alterations in peptides originating from non‐collagen proteins. We specifically focused on proteins represented by peptides that showed a consistent directional change in abundance. Among proteins represented by peptides increased in patients with HF, several proteins present in blood were found, including beta‐2‐microglobulin (a component of the major histocompatibility antigen indicating activation of the immune system and widely considered to reflect renal tubular function), 35 kDa inter‐alpha‐trypsin inhibitor heavy chain H4 (an acute‐phase protein participating in inflammatory responses to trauma), apolipoprotein C‐III (involved in triglyceride homeostasis), and matrix Gla protein (an inhibitor of vascular mineralization involved in bone organization). Excretion of proteins in urine may reflect not only localized kidney disease, but also vascular endothelial dysfunction.^24^ As kidney function was adjusted for in the case‐control matching, increased abundance of peptides originating from plasma proteins may indicate vascular endothelial dysfunction, known to contribute to the development of HF.^25^

Besides pathways reflecting alteration in collagen turnover (such as collagen degradation, activation of matrix metalloproteinases, anchoring fibril formation), the second most prominent finding was pathways associated with immune response, mostly innate immunity. Our analysis revealed alterations in Toll‐like receptor (TLR) cascades, the involvement of which in the inflammatory response in HF is well recognised.^26^ TLR signalling triggers inflammation leading to maladaptive wound healing and fibrosis. Besides impact of HF on TLRs, urinary proteomics data also indicate dysregulation of pathways representing effector systems of the innate immunity, including neutrophil degranulation and complement cascade. Neutrophils play a role in the early phase of cardiac healing upon myocardial injury through clearance of dead cells and polarisation of macrophages, while neutrophil dysregulation leads to heart dysfunction and failure.^27^ Abnormal complement activation promotes inflammation and tissue damage, and has been reported in patients with HF.^28^

Surprisingly, the three HF subtypes could not be separated based on urinary peptide analysis, whereas the differences between patients with HF and non‐HF controls were obvious. This implies that the molecular alterations in urine are indifferent to the EF. In line with our findings, it has been proposed that changes in fibrillar collagen (collagen type I) turnover are common in HF independent of EF.^21^ This suggests that EF, although a cornerstone in HF management, may not be related to specific molecular changes, further supporting concerns about the validity of the current definitions of HF subtypes.^29^ Our findings may also help explain why developing effective therapies for certain HF subtypes using EF has proved challenging. It may be worth investigating the potential of subtyping HF patients based on molecular alterations, analogous to the approach established in oncology.^30^ Characterisation and subtyping of HF through the clustering of urinary data (alone or as a part of multi‐omics approach) may enable more precise definition of disease subtypes, leading to the development of more effective drugs for sub‐populations of patients and thus personalised treatment. Although the number of urinary peptides that differed significantly from controls was highest amongst patients with HFrEF, 84% of peptides differing between HFmrEF and controls, and 78% differing between HFpEF and controls overlapped with those differing between HFrEF and controls, with similar quantitative changes. The difference in numbers of significant peptides is likely due to the smaller number of patients in the HFmrEF and HFpEF groups. The increase in the number of significant markers with increasing samples size has been described previously, with the plateau reached at sample sizes around *n* = 800.^16^

Collectively, considering the complex pathophysiology underlying HF, urinary proteomics may have an advantage to better profile fibrosis and also systemic impact of HF in comparison to single biomarkers. Based on urinary peptide alterations, it appears that targeting collagen degradation may be feasible and a novel approach to combat fibrosis. Further studies are planned to evaluate the applicability of the biomarkers we have identified for detection of patients with HF. One aim is investigating if the biomarkers identified here would enable early detection of HF, prior to the onset of symptoms, to enable timely (ideally preventive) intervention. In addition, we aim at testing the value of the biomarkers described here in predicting disease progression. Further, testing of the biomarkers for their value in predicting drug response, as was e.g. shown for CKD273,^31^ seems a valid approach. Based on these results the ultimate goal is application of the findings to stratify patients in clinical trials based on their proteomics profile to target treatment. The use of the urinary proteomic data alone, or as a part of multi‐omics phenotyping, may ultimately lead to better characterization of disease pathophysiology and may help identifying targets for novel therapeutic strategies.

### Study strengths and limitations

The strength of our study was a large sample size covering patients with HF across the full range of EF. As a result, confidence in the validity of the findings is very high. Moreover, all urinary peptide datasets were acquired using the same analytical platform (CE‐MS), with analysis and data processing performed according to ISO13485 standards. This allows for generation of highly comparable data, enabling comparisons in the wide spectrum of conditions.

Limitations included that individual patient data were derived from the different cohorts of HF patients and controls enrolled at multiple clinical centres. However, case‐control matching for clinical/demographical variables known to have an impact on urinary peptides allowed to harmonise the data in the respective comparisons. Although efforts were made to collect urine samples on the same day as other study assessments, this may not have been the case for every participant. There was no standard operating procedure applied for the collection of all urine samples, specifically there were no requirements made for an early morning or mid‐stream sample. However, previous studies based on CE‐MS have shown good reproducibility of data, even if sampling is not fully standardized.^3^, ^4^ On the other hand, inclusion of patients from different cohorts can also be seen as a strength, as our findings were consistent across different cohorts.

In addition, this was a retrospective cross‐sectional analysis. Therefore, for non‐HF controls, the information on measurements performed for patients suspicious for HF (e.g. NT‐proBNP, BNP, EF, etc.) is frequently missing. Our study was not designed to compare the diagnostic performance of urinary peptides with NT‐proBNP and BNP. Those plasma biomarkers were used as one of the criteria for defining HF and thus inclusion/exclusion of patients in our study, preventing an unbiased comparison. A previous study demonstrated that a set of urinary peptides had similar utility as BNP in diagnosing patients with HF, while a combination of BNP with urinary peptides provided additional diagnostic information.^7^ Information about medications (e.g. angiotensin‐converting enzyme inhibitors, angiotensin receptor–neprilysin inhibitor) was not available for all cohorts, and thus was not taken into account. Similar applies to information on disease aetiology (ischaemic or non‐ischaemic) and thus, those patient groups were not discriminated in our study. Moreover, associations between urinary markers and clinical outcomes were not investigated. However, in a recent study the value of urinary collagen type I peptides in predicting outcome in HF has been demonstrated.^32^

## Conclusions

Heart failure is consistently associated with differences in urine peptides, compared to non‐HF controls, reflecting fibrosis and the systemic impact of HF. HF subtypes based on left ventricular phenotype, although clinically different, are very similar at the level of urinary peptides. Definition of HF subtypes based on molecular changes, similar to oncology, may be more appropriate, especially when aiming for personalized intervention. Application of urinary peptides to support management of patients with HF, although feasible, requires further assessment in prospective trials, to investigate the potential for early detection, molecular subtyping, prediction of therapeutic response as well as guiding therapeutic intervention.

### Funding

AC, CD, JD, AG, AM, JR, NG, PR, FZ, HM, ZZ, JS, AL are supported by the EU Commission via the HOMAGE (HEALTH‐FP7–305507 HOMAGE) project. CD, FZ, HM, JD and JS were previously supported by the EU‐MASCARA (HEALTH‐FP7–278249) project, and CD and JM receive support from the British Heart Foundation (RE/18/6/34217). TH is supported by the EU Commission via the CaReSyAn Project (MSCA‐ITN‐2017‐Project ID: 764474). VJ is supported by the Deutsche Forschungsgemeinschaft' (DFG, German Research Foundation) by the Transregional Collaborative Research Centre (TRR 219; Project‐ID 322900939) (subproject S‐03). PR, NG, FZ are supported by the RHU Fight‐HF, a public grant overseen by the French National Research Agency (ANR) as part of the second ‘Investissements d'Avenir’ program (reference: ANR‐15‐RHUS‐0004), and by the French PIA project ‘Lorraine Université d'Excellence’(reference: ANR‐15‐IDEX‐04‐LUE). The Stanislas Cohort is sponsored by Nancy CHRU (France); its biobanking is managed by Biological Resource Center Lorrain BB‐0033‐00035. Generation Scotland received core support from the Chief Scientist Office of the Scottish Government Health Directorates [CZD/16/6] and the Scottish Funding Council [HR03006] and is currently supported by the Wellcome Trust [216767/Z/19/Z]. The non‐profit Research Institute Alliance for the Promotion of Preventive Medicine received a non‐binding grant from OMRON Healthcare Co., Inc., Kyoto, Japan.

**Conflict of interest:** HM, AV, JS and VJ are members of the European Uraemic Toxin Working Group (EUTox). HM is cofounder and co‐owner of Mosaiques Diagnostics. TH, MM, JR, JS and AL are employees of Mosaiques Diagnostics GmbH. PR reports personal fees (consulting) for Idorsia,G3P, KBP, and Sanofi, honoraria from AstraZeneca, Bayer, Boehringer Ingelheim, CVRx, Fresenius, Grunenthal, Novartis, NovoNordisk, Sequana medical, Servier, Stealth Peptides, Ablative Solutions, Corvidia, Relypsa and Vifor Fresenius Medical Care Renal Pharma, outside the submitted work, PR is the cofounder of CardioRenal. NG reports personal fees from Novartis, AstraZeneca, Bayer, Boehringer, Vifor, outside the submitted work. FZ reports personal fees from Janssen, Bayer, Amgen, CVRx, Boehringer, AstraZeneca, Cardior, Cereno pharmacuetical, Applied Therapeutics, Merck, Novartis, NovoNordisk, Myokardia, Actelion, Owkin, Cellprothera, other from CVCT and Cardiorenal, outside the submitted work. AM reports personal fees from Orion, Servier, Otsuka, Philips, Sanofi, Adrenomed, Epygon and Fire 1 and grants and personal fees from 4TEEN4, Abbott, Roche and Sphyngotec. JM reported other support from AstraZeneca; non‐financial support from Cytokinetics, Bayer, Theracos, Oxford University, Dalcor, Merck, GlaxoSmithKline, Bristol Myers Squibb, Vifor‐Fresenius, Kidney Research UK, Alnylam, Abbvie, Cyclerion, Cardurion; and personal fees from Amgen, and personal fees from Abbott, Hickma, Sun Pharmaceuticals, Servier lecture fees outside the submitted work.

## Supporting information


**Appendix S1.** Supplementary Methods. Detailed information on CE‐MS analysis, peptide sequencing and bioinformatics.
**Table S1.** Characteristics of the matched heart failure patients. Calculations are presented as median [interquartile range] or number (%). Bold indicates *P* < 0.05. Mann–Whitney test was used for continuous variables, while the Chi‐squared test was applied for categorical variables.
**Table S2.** Discriminatory metrics for the selected urinary biomarkers. Information is given for the top 20 peptides providing the greatest discrimination between patients with HF (*n* = 773) and matched controls (*n* = 773) as well as between matched controls and patients with HFrEF (*n* = 442), HFmrEF (*n* = 144), HFpEF (*n* = 187) (as listed in *Table* *2*). Average abundance, fold change, *P*‐value (BH adjusted), frequency, AUC, sensitivity, specificity, PPV and NPV are provided. Sensitivity and specificity values are given at the optimum cut‐off level that was defined to maximise both sensitivity and specificity values.
**Table S3.** Distribution of peptides by original cohort. Analysis was performed for the three most representative heart failure cohorts with the highest number of patients [i.e. Campbell *et al*. 2020 (*n* = 449), Futter *et al*. 2011 (*n* = 231), and Rossing *et al*. 2016 (*n* = 91)]. Information is given for the top 20 peptides providing the greatest discrimination between patients with HF (*n* = 773) and controls (*n* = 773). Average abundance, fold change and frequency in each analysed cohort are given. Differences in peptide abundance between three groups was evaluated using Kruskal–Wallis test, followed by post‐hoc analysis using Dunn test with Bonferroni method for *P*‐value adjustment.
**Table S4.** Distribution of peptides in patients enrolled with acute (*n* = 89) and chronic heart failure (*n* = 682). Information is given for top 20 peptides providing the greatest discrimination between patients with HF (*n* = 773) and controls (*n* = 773). Average abundance, fold change and frequency in each analysed group are given. Peptide abundance between three groups was evaluated using Kruskal–Wallis test, followed by post‐hoc analysis using Dunn test with Bonferroni method for *P*‐value adjustment.
**Table S5.** Urinary peptide differences between heart failure subtypes. Significantly altered peptides between HFrEF (*n* = 117) and HFpEF (*n* = 117) matched for sex, age, eGFR, systolic and diastolic blood pressure, diabetes and hypertension are listed, along with peptide characteristics (mass, retention time, sequence), frequency, average abundance and *P*‐value (BH adjusted). Discriminatory metrics i.e. AUC, sensitivity, specificity, PPV and NPV are given. Significant peptides were defined as passing the criterion of *P* < 0.05 and the frequency threshold of 30%. Peptides are sorted by increasing *P*‐value.
**Table S6.** List of the shortlisted *in silico* predicted proteases. Proteases that did not meet the criteria for shortlisting (i.e. at least one protease/CS association reported in the literature and the percentage of cleavage events above 1%) are marked in grey.

**Figure S1.** Workflow for matching patients with heart failure. Participants diagnosed with HFrEF (*n* = 442), HFmrEF (*n* = 144), HFpEF (*n* = 187) were matched for sex, age, eGFR, systolic and diastolic blood pressure, diabetes and hypertension. This resulted in the selection of 117 individuals in each group. ^*^When performing matching for patients with HFmrEF and HFpEF, HFmrEF patients that have been matched to HFrEF were considered. ^**†**^When performing matching for patients with HFrEF and HFpEF, HFrEF patients that have been matched to HFmrEF were considered, similarly ^**‡**^HFpEF that have been matched to HFmrEF were considered.

**Figure S2.** Analysis of heart failure patients stratified by cohort. Analysis was performed for three cohorts with the highest number of HF patients [i.e. Campbell *et al*. 2020 (*n* = 449), Futter *et al*. 2011 (*n* = 231), and Rossing *et al*. 2016 (*n* = 91)]. Results are provided for peptides found to be significantly different between all patients with HF (*n* = 773) and controls (*n* = 773) including 577 peptides (*A* and *B*) and, separately, the 20 peptides with the greatest discrimination between HF and controls (*C*). Correlation of (*A*) the peptide fold changes calculated in selected cohorts and in the complete HF cohort (*n* = 773), in comparison to all controls included in the study and (*B*) average peptide abundance in three HF cohorts. (*C*) Box‐plots displaying peptide abundance per cohort. Peptide abundance for the controls (*n* = 773) is provided as a comparator. Mean is indicated with a red diamond.

**Figure S3.** Analysis of heart failure patients stratified based on the enrolment status (acute and chronic heart failure). (*A*) Correlation of peptides significantly different between HF and controls when comparing average peptide abundance observed in patients with acute (*n* = 89) and chronic HF (*n* = 682). (*B*) Distribution of abundance for top 20 peptides exhibiting greatest discrimination between HF and controls (*Table* *2*) in patients with acute and chronic HF and controls. Mean is indicated with a red diamond.

**Figure S4.** Comparison of chronic kidney disease and heart failure associated urinary peptides. (*A*) The comparison was performed between 273 CKD associated peptides defined previously when comparing patients with CKD and normal controls (Good *et al*., 2010) and 577 HF associated peptides defined in this study. Seventy‐one peptides were found overlapping between these two sets of biomarkers. (*B*) Correlation analysis of fold changes for 71 common peptides is presented.

**Figure S5.** Summary of the consensus clustering results. Segregation of patients with HF only into clusters for k = 2–8 solutions. Percentage of patients assigned to the class is given. Chi‐squared test was applied to assess differences in the distribution of patients within the class.

**Figure S6.** Performance of biomarker panels discriminating between heart failure subtypes. The urinary peptidomics data from patients with HFrEF (*n* = 117), HFmrEF (*n* = 117) and HFpEF (*n* = 117) matched for sex, age, eGFR, SBP, DBP, diabetes and hypertension, were randomly divided into two sets (training set, *n* = 70 and test set, *n* = 47). Thirty peptides with the highest AUC were selected in the training set (in each pairwise analysis separately) and combined using SVM, followed by optimisation of SVM parameters. Performance of biomarkers was assessed in the test set. Receiving operating characteristic analysis based on test set data was conducted for combination of biomarkers discriminating (*A*) patients with HFrEF (*n* = 47) from patients with HFmrEF (*n* = 47), (*B*) patients with HFmrEF (*n* = 47) from patients with HFpEF (*n* = 47) and (*C*) patients with HFrEF (*n* = 47) from patients with HFpEF (*n* = 47). Information on specificity and sensitivity of the model at the pre‐specified cut‐off (based on the Youden index J) is provided.

**Figure S7.** Urinary peptides differences between heart failure subtypes and controls. Volcano plot showing distribution of the identified sequenced peptides between matched controls and patients with HFrEF, HFmrEF and HFpEF. Directionality of the difference, magnitude as well as significance level (BH adjusted *P*‐value) are displayed. Discrimination between collagen and non‐collagen derived peptides is provided. Peptides originated from proteins for which at least 10 significant peptides were identified (*P* < 0.05, BH adjusted) when comparing all patients with HF and controls are color‐coded. Peptides with *P* < 0.05 (BH adjusted) are marked in grey.
