# Supplementary material for: Urinary peptides in heart failure: a link to molecular pathophysiology
Source: Eur J Heart Fail. 2021 May 7;23(11):1875–87. doi: 10.1002/ejhf.2195 (PMC9291452; doi:10.1002/ejhf.2195)
Supplement: Supplementary file 1 — Appendix S1. Supplementary Methods. Detailed information on CE‐MS analysis, peptide sequencing and bioinformatics. Table S1. Characteristics of the matched heart failure patients. Calculations are presented as median [interquartile range] or number (%). Bold indicates P < 0.05. Mann–Whitney test was used for continuous variables, while the Chi‐squared test was applied for categorical variables. Table S2. Discriminatory metrics for the selected urinary biomarkers. Information is given for the top 20 peptides providing the greatest discrimination between patients with HF (n = 773) and matched controls (n = 773) as well as between matched controls and patients with HFrEF (n = 442), HFmrEF (n = 144), HFpEF (n = 187) (as listed in Table 2 ). Average abundance, fold change, P‐value (BH adjusted), frequency, AUC, sensitivity, specificity, PPV and NPV are provided. Sensitivity and specificity values are given at the optimum cut‐off level that was defined to maximise both sensitivity and specificity values. Table S3. Distribution of peptides by original cohort. Analysis was performed for the three most representative heart failure cohorts with the highest number of patients [i.e. Campbell et al. 2020 (n = 449), Futter et al. 2011 (n = 231), and Rossing et al. 2016 (n = 91)]. Information is given for the top 20 peptides providing the greatest discrimination between patients with HF (n = 773) and controls (n = 773). Average abundance, fold change and frequency in each analysed cohort are given. Differences in peptide abundance between three groups was evaluated using Kruskal–Wallis test, followed by post‐hoc analysis using Dunn test with Bonferroni method for P‐value adjustment. Table S4. Distribution of peptides in patients enrolled with acute (n = 89) and chronic heart failure (n = 682). Information is given for top 20 peptides providing the greatest discrimination between patients with HF (n = 773) and controls (n = 773). Average abundance, fold change and frequency in each [file EJHF-23-1875-s002.pdf]

European Journal of Heart Failure

Supporting Information

This appendix formed part of the original submission and has been peer-reviewed.

Supplement to: Urinary peptides in heart failure: a link to molecular pathophysiology. European Journal of Heart Failure 2021.

Table of contents

Table S1. Characteristics of the matched HF patients. 2

Table S2. Discriminatory metrics for the selected urinary biomarkers. 4

Table S3. Distribution of peptides by original cohort. 8

Table S4. Distribution of peptides in patients enrolled with acute (n=89) and chronic HF (n=682). .. 10

Table S5. Urinary peptide differences between HF subtypes. 12

Table S6. List of the shortlisted *in silico* predicted proteases. 14

Figure S1. Workflow for matching patients with HF. 15

Figure S2. Analysis of HF patients stratified by cohort. 16

Figure S3. Analysis of HF patients stratified based on the enrolment status (acute and chronic HF). 18

Figure S4. Comparison of CKD and HF associated urinary peptides. 19

Figure S5. Summary of the consensus clustering results. 20

Figure S6. Performance of biomarker panels discriminating between HF subtypes. 21

Figure S7. Urinary peptides differences between HF subtypes and controls. 22

Supplementary methods. 23

CE-MS 23

Sequencing of peptides 23

Bioinformatic analysis 24

Table S1. Characteristics of the matched HF patients. Calculations are presented as median [Interquartile Range] or number (%). Bold indicates $p < 0.05$. Mann-Whitney test was used for continuous variables, while the Chi-squared test was applied for categorical variables.

Characteristics	HFrEF N=117	HFmrEF N=117	HFpEF N=117	*p*-value
Age - yr	70.00 [63.00-75.50]	68.00 [63.75-73.00]	70.00 [63.75- 75.00]	* *P* = 0.1881<br>† *P* = 0.2477<br>‡ *P* = 0.6948
Female sex – no. (%)	50 (42.7)	40 (34.2)	48 (41.0)	* *P* = 0.2265<br>† *P* = 0.3448<br>‡ *P* = 0.8946
Systolic BP – mmHg	136.00 [125.75-152.25]	135.00 [123.75- 151.50]	135.00 [121.00- 150.75]	* *P* = 0.8582<br>† *P* = 0.4205<br>‡ *P* = 0.3222
Diastolic BP – mm Hg	75.00 [67.00-83.00]	76.00 [68.00-82.00]	72.00 [64.75- 82.00]	* *P* = 0.5256<br>† *P* = 0.0972<br>‡ *P* = 0.3374
Heart rate – beats/min	73.00 [64.75-86.00]	71.00 [60.00-80.00]	69.00 [60.00-80.00]	* *P* = 0.0700<br>† *P* = 0.6360<br>‡ ***P* = 0.0256**
Body mass index – kg/m²	28.69 [24.03-31.89]	29.62 [26.26-35.33]	31.63 [27.67-36.07]	* ***P* = 0.0228**<br>† *P* = 0.0999<br>‡ ***P* = 0.0002**
eGFR – ml/min/1.73m² §	64.11 [46.05-82.35]	65.69 [53.62-83.69]	65.48 [49.39-79.30]	* *P* = 0.1953<br>† *P* = 0.4273<br>‡ *P* = 0.7173
EF – %	30.00 [23.75- 35.00]	43.00 [41.00-46.00]	55.00 [53.00-62.00]	* ***P* < 0.0001**<br>† ***P* < 0.0001**<br>‡ ***P* < 0.0001**
NT-proBNP – pg/mL	969.00 [405.00-1874.70]	482.00 [301.00-819.00]	658.00 [317.00- 1109.00]	* ***P* = 0.0116**<br>† *P* = 0.2218<br>‡ *P* = 0.0590
BNP – pg/mL	480.00 [230.00-1277.50]	218.00 [128.00-429.00]	234.00 [141.75-465.75]	* ***P* = 0.0002**<br>† *P* = 0.5782<br>‡ ***P* = 0.0019**
NYHA functional class – no. (%)				
0	3 (2.6)	1 (0.8)	0 (0.0)	* ***P* = 0.0001**
I	11 (9.4)	24 (20.5)	27 (23.1)	† *P* = 0.5451
II	56 (47.9)	76 (65.0)	67 (57.3)	‡ ***P* = 0.0008**
III	43 (36.8)	15 (12.9)	21 (17.9)	
IV	4 (3.4)	1 (0.8)	2 (1.7)	
Hypertension, no. (%)	78 (66.7)	77 (65.8)	69 (59.0)	* *P* = 1.0000<br>† *P* = 0.3448<br>‡ *P* = 0.2792
Diabetes, no. (%)	51 (43.6)	43 (36.8)	52 (44.4)	* *P* = 0.3506<br>† *P* = 0.2869<br>‡ *P* = 1.0000
CAD, no. (%)	65 (55.6)	53 (45.3)	48 (41.0)	* *P* = 0.1504<br>† *P* = 0.5975<br>‡ ***P* = 0.0363**
CKD, no. ǁ	52 (44.4)	48 (41.0)	49 (41.9)	* *P* = 0.6918<br>† *P* = 1.0000<br>‡ *P* = 0.7918

Abbreviations: **BNP**=brain natriuretic peptide, **BP**= blood pressure, **CAD**= coronary artery disease, **CKD**= chronic kidney disease, **eGFR=** estimated glomerular filtration rate, **EF**= ejection fraction, **HF**= heart failure, **HFmrEF**= HF with mid-range ejection fraction, **HFpEF**= HF with preserved ejection fraction, **HFrEF**= HF with reduced ejection fraction, **NT-proBNP**= N-terminal prohormone of B-type natriuretic peptide, **NYHA**= New York Heart Association

*****HFrEF versus HFmrEF, [†]HFmrEF versus HFpEF, [‡]HFrEF versus HFpEF, [§]eGFR was estimated based on Chronic Kidney Disease Epidemiology Collaboration (CKD-EPI) equation, [‖]CKD was defined based on the clinical diagnosis and/or on the kidney function (eGFR<60 ml/min/$1.73m^2$).

Table S2. Discriminatory metrics for the selected urinary biomarkers. Information is given for the top 20 peptides providing the greatest discrimination between patients with HF (n=773) and matched controls (n=773) as well as between matched controls and patients with HFrEF (n=442), HFmrEF (n=144), HFpEF (n=187) (as listed in **Table 2**). Average abundance, fold change, *p*-value (BH adjusted), frequency, AUC, sensitivity, specificity, PPV and NPV are provided. Sensitivity and specificity values are given at the optimum cut-off level that was defined to maximise both sensitivity and specificity values.

ID	HF (n=773) versus Controls (n=773)										
	Fold change	*p*-value (BH)	Avg. HF	Avg. Controls	Freq. HF, n (%)	Freq. Controls, n (%)	AUC	Sensitivity	Specificity	PPV	NPV
e00005	3.96	6.75E-69	129.93	32.81	547 (71)	220 (28)	0.75	0.60	0.86	0.81	0.68
e01132	1.93	2.92E-51	7882.95	4076.58	772 (100)	758 (98)	0.73	0.75	0.60	0.65	0.70
e00340	2.12	5.12E-49	1025.07	483.00	752 (97)	616 (80)	0.72	0.84	0.49	0.62	0.75
e08489	3.00	1.23E-46	372.63	124.19	622 (80)	405 (52)	0.71	0.58	0.75	0.70	0.64
e00421	1.93	6.38E-46	958.89	497.49	726 (94)	649 (84)	0.72	0.65	0.70	0.69	0.67
e01461	2.70	7.60E-46	1335.60	494.01	539 (70)	274 (35)	0.70	0.67	0.69	0.68	0.68
e00152	2.94	1.22E-45	89.27	30.39	480 (62)	202 (26)	0.70	0.62	0.74	0.71	0.66
e16278	0.27	8.05E-45	124.00	452.62	411 (53)	577 (75)	0.71	0.54	0.81	0.74	0.64
e00274	4.15	3.33E-44	46.91	11.30	426 (55)	165 (21)	0.69	0.54	0.81	0.74	0.64
e05864	0.33	4.82E-44	713.84	2158.39	577 (75)	659 (85)	0.71	0.49	0.86	0.78	0.63
e00134	3.38	3.61E-39	26.99	7.98	399 (52)	152 (20)	0.67	0.52	0.80	0.73	0.62
e15360	0.32	1.74E-38	399.29	1229.79	714 (92)	711 (92)	0.70	0.51	0.81	0.74	0.63
e03354	1.64	1.91E-37	8444.25	5157.35	758 (98)	732 (95)	0.70	0.62	0.74	0.70	0.66
e00957	2.72	1.13E-36	51.55	18.93	390 (50)	150 (19)	0.66	0.48	0.84	0.75	0.62
e05726	0.18	1.78E-36	57.53	314.80	268 (35)	475 (61)	0.68	0.50	0.79	0.71	0.61
e04294	3.09	6.60E-34	80.44	26.01	333 (43)	102 (13)	0.65	0.43	0.87	0.77	0.60
e00635	10.43	9.92E-34	122.96	11.79	397 (51)	156 (20)	0.66	0.51	0.80	0.72	0.62

**e00131**	3.21	1.23E-30	64.76	20.20	392 (51)	174 (23)	0.65	0.51	0.78	0.70	0.61
**e01767**	2.63	1.43E-30	94.80	36.03	454 (59)	251 (32)	0.66	0.52	0.77	0.70	0.62
**e00259**	1.57	2.61E-30	223.27	142.16	725 (94)	534 (69)	0.67	0.86	0.47	0.62	0.76

**ID**	**HFrEF (n=442) versus Controls (n=442)**										
	**Fold change**	***p*-value (BH)**	**Avg. HFrEF**	**Avg. Controls**	**Freq. HFrEF, n (%)**	**Freq. Controls, n (%)**	**AUC**	**Sensitivity**	**Specificity**	**PPV**	**NPV**
**e00005**	3.54	8.13E-39	128.37	36.27	325 (74)	133 (30)	0.75	0.67	0.80	0.77	0.70
**e08489**	3.35	4.21E-34	437.97	130.76	367 (83)	229 (52)	0.75	0.66	0.75	0.73	0.69
**e05864**	0.29	2.03E-31	607.31	2127.86	325 (74)	381 (86)	0.74	0.54	0.87	0.81	0.65
**e00421**	1.9	2.44E-28	980.35	514.78	421 (95)	369 (83)	0.73	0.76	0.64	0.68	0.72
**e00274**	3.94	5.94E-26	45.96	11.66	253 (57)	94 (21)	0.69	0.56	0.81	0.75	0.65
**e00152**	2.57	9.78E-26	89.74	34.91	286 (65)	119 (27)	0.70	0.64	0.75	0.72	0.67
**e00134**	3.64	2.15E-25	28.01	7.69	236 (53)	82 (19)	0.69	0.53	0.82	0.75	0.63
**e01461**	2.43	4.12E-25	1388.28	570.69	321 (73)	166 (38)	0.70	0.70	0.67	0.68	0.69
**e05726**	0.14	6.46E-25	45.44	313.98	146 (33)	277 (63)	0.70	0.60	0.74	0.70	0.65
**e03354**	1.66	6.62E-25	8773.73	5272.14	436 (99)	416 (94)	0.71	0.66	0.72	0.70	0.68
**e01132**	1.82	4.80E-24	7391.60	4071.86	441 (100)	434 (98)	0.71	0.72	0.61	0.65	0.68
**e00340**	1.94	4.86E-24	920.49	474.94	431 (98)	349 (79)	0.71	0.64	0.67	0.66	0.65
**e16278**	0.29	1.31E-21	128.62	449.47	241 (55)	321 (73)	0.69	0.55	0.80	0.73	0.64
**e00957**	2.28	2.04E-21	52.80	23.13	239 (54)	96 (22)	0.67	0.50	0.83	0.75	0.63
e08350	2.98	5.42E-21	115.71	38.87	257 (58)	117 (26)	0.68	0.54	0.79	0.72	0.63
**e15360**	0.32	3.18E-20	385.75	1199.27	408 (92)	398 (90)	0.69	0.50	0.84	0.76	0.63
**e04294**	2.92	6.80E-20	89.91	30.81	203 (46)	63 (14)	0.65	0.46	0.86	0.77	0.61
**e01767**	2.53	2.37E-19	102.76	40.55	271 (61)	141 (32)	0.67	0.56	0.75	0.69	0.63
**e09631**	3.79	9.86E-19	306.57	80.83	182 (41)	58 (13)	0.64	0.41	0.88	0.77	0.60
e02530	1.91	1.66E-17	623.12	326.96	359 (81)	278 (63)	0.68	0.62	0.70	0.68	0.65

**ID**	**HFmrEF (n=144) versus Controls (n=144)**										
	**Fold change**	***p*-value (BH)**	**Avg. HFmrEF**	**Avg. Controls**	**Freq. HFmrEF, n (%)**	**Freq. Controls, n (%)**	**AUC**	**Sensitivity**	**Specificity**	**PPV**	**NPV**

**e00005**	4.89	2.21E-12	131.66	26.93	102 (71)	39 (27)	0.76	0.63	0.88	0.84	0.70
**e01132**	2.01	8.53E-12	7301.92	3629.68	144 (100)	140 (97)	0.77	0.69	0.76	0.75	0.71
**e01461**	4.30	5.43E-10	1365.55	317.94	102 (71)	53 (37)	0.74	0.58	0.88	0.83	0.67
e11533	0.26	5.43E-10	161.12	611.02	73 (51)	117 (81)	0.75	0.63	0.78	0.74	0.67
**e00152**	5.17	1.83E-08	90.97	17.58	86 (60)	37 (26)	0.71	0.52	0.87	0.81	0.64
e00153	5.73	5.07E-08	86.20	15.03	76 (53)	27 (19)	0.69	0.50	0.88	0.81	0.63
**e00340**	2.03	5.17E-08	967.78	475.83	138 (96)	118 (82)	0.72	0.81	0.56	0.65	0.74
**e00421**	1.99	6.53E-08	947.30	475.40	134 (93)	125 (87)	0.72	0.59	0.77	0.73	0.65
**e00131**	3.98	8.58E-08	75.78	19.04	82 (57)	31 (22)	0.69	0.56	0.79	0.74	0.64
**e00274**	4.20	9.83E-08	49.67	11.83	81 (56)	33 (23)	0.69	0.49	0.87	0.80	0.63
**e00957**	3.81	1.19E-07	56.12	14.73	79 (55)	29 (20)	0.69	0.50	0.88	0.81	0.63
e15365	0.15	1.19E-07	34.38	233.21	52 (36)	94 (65)	0.70	0.61	0.74	0.71	0.65
**e15360**	0.31	1.24E-07	400.89	1301.42	130 (90)	134 (93)	0.72	0.55	0.85	0.80	0.65
**e00635**	8.62	5.53E-07	83.85	9.73	76 (53)	27 (19)	0.68	0.52	0.83	0.76	0.63
**e05726**	0.18	1.22E-06	57.30	326.91	48 (33)	88 (61)	0.69	0.58	0.76	0.72	0.64
**e09631**	6.78	1.37E-06	277.82	40.98	57 (40)	15 (10)	0.65	0.37	0.92	0.84	0.59
**e16278**	0.23	1.52E-06	115.63	492.83	81 (56)	107 (74)	0.70	0.55	0.80	0.74	0.64
**e02352**	2.74	2.52E-06	107.44	39.27	80 (56)	30 (21)	0.67	0.55	0.80	0.74	0.64
**e03354**	1.66	3.10E-06	8531.45	5154.94	141 (98)	138 (96)	0.70	0.58	0.81	0.76	0.65
e06138	7.00	3.36E-06	95.79	13.69	48 (33)	10 (7)	0.64	0.33	0.94	0.87	0.58

ID	HFpEF (n=187) versus Controls (n=187)										
	**Fold change**	***p*-value (BH)**	**Avg. HFpEF**	**Avg. Controls**	**Freq. HFpEF, n (%)**	**Freq. Controls, n (%)**	**AUC**	**Sensitivity**	**Specificity**	**PPV**	**NPV**
**e00340**	2.59	1.80E-14	1316.37	507.57	183 (98)	149 (80)	0.76	0.57	0.83	0.77	0.65
**e16278**	0.28	3.00E-14	119.54	429.09	89 (48)	149 (80)	0.75	0.71	0.73	0.73	0.71
**e00005**	4.53	3.78E-13	132.29	29.17	120 (64)	48 (26)	0.73	0.57	0.89	0.84	0.67
**e01132**	2.14	7.49E-13	9491.74	4431.87	187 (100)	184 (98)	0.75	0.63	0.74	0.71	0.66
**e00635**	11.88	2.13E-10	93.29	7.86	98 (52)	30 (16)	0.69	0.52	0.86	0.79	0.64
**e00152**	2.94	1.71E-08	86.85	29.57	108 (58)	46 (25)	0.68	0.58	0.75	0.71	0.64

**e00259**	1.74	3.26E-08	242.03	139.23	179 (96)	129 (69)	0.70	0.89	0.47	0.63	0.80
**e01461**	2.65	3.26E-08	1188.04	448.38	116 (62)	55 (29)	0.68	0.60	0.74	0.70	0.65
e18263	0.16	4.10E-08	6.19E+01	3.87E+02	24 (13)	79 (42)	0.65	0.42	0.89	0.80	0.60
**e15360**	0.34	5.46E-08	430.06	1246.78	176 (94)	179 (96)	0.69	0.83	0.45	0.60	0.72
**e00274**	4.69	6.17E-08	47.04	10.03	92 (49)	38 (20)	0.66	0.47	0.87	0.79	0.62
e02641	8.55	7.16E-08	173.40	20.29	66 (35)	15 (8)	0.64	0.35	0.92	0.83	0.59
**e00421**	1.94	1.91E-07	917.09	473.63	171 (91)	155 (83)	0.69	0.58	0.73	0.69	0.63
e01453	2.45	1.96E-07	49.32	20.15	108 (58)	48 (26)	0.67	0.57	0.75	0.70	0.63
**e00134**	2.76	2.67E-07	26.19	9.49	91 (49)	35 (19)	0.66	0.48	0.82	0.73	0.61
**e08489**	2.97	5.72E-07	285.11	95.91	141 (75)	98 (52)	0.68	0.64	0.66	0.66	0.64
e00637	2.64	6.01E-07	242.81	91.90	138 (74)	98 (52)	0.67	0.59	0.71	0.68	0.63
e03389	0.47	1.09E-06	1154.04	2446.40	155 (83)	162 (87)	0.68	0.53	0.78	0.71	0.62
e07917	2.69	1.09E-06	155.05	57.73	108 (58)	56 (30)	0.66	0.55	0.74	0.69	0.62
**e02352**	3.14	1.34E-06	133.52	42.51	86 (46)	35 (19)	0.65	0.43	0.84	0.74	0.59

Abbreviations: AUC = area under the receiver operating characteristic curve, **Avg. abundance** = average peptide abundance, **BH**=Benjamini-Hochberg, **Freq.**= frequency, **HF**= heart failure, **HFmrEF**= heart failure with mid-range ejection fraction, **HFpEF**= heart failure with preserved ejection fraction, **HFrEF**= heart failure with reduced ejection fraction, **NPV** = negative predictive value, **PPV** = positive predictive value.

Peptides are ordered by increasing *p*-value in respective comparisons.
Underscore indicates overlapping peptides between presented comparisons.
Peptides higher in disease are labeled in green, lower in red.

Table S3. Distribution of peptides by original cohort. Analysis was performed for the three most representative HF cohorts with the highest number of patients (i.e. Campbell *et al.* 2020 (n= 449), Futter *et al.* 2011 (n=231), and Rossing *et al*. 2016 (n=91)). Information is given for the top 20 peptides providing the greatest discrimination between patients with HF (n=773) and controls (n=773). Average abundance, fold change and frequency in each analysed cohort are given. Differences in peptide abundance between three groups was evaluated using Kruskal-Wallis test, followed by post-hoc analysis using Dunn test with Bonferroni method for *p*-value adjustment.

	**Avg. abundance**				**Fold change**				**Frequency**					**p-value (post hoc test)**					
**Peptide ID**	HF - Campbell *et al.* 2020 (n= 449)	HF - Futter *et al.* 2011 (n=231)	HF - Rossing *et al.* 2016 (n=91)	Controls (n=773)	HF- Campbell *et al.* 2020 vs Controls	HF- Futter *et al.* 2011 vs Controls	HF- Rossing *et al.* 2016 vs Controls	HF (n=773) vs Controls (n=773)	HF - Campbell *et al.* 2020, n (%)	HF- Futter *et al.* 2011, n (%)	HF – Rossing *et al.* 2016, n (%)	Controls, n (%)	*p*-value (Kruskal-Wallis)	HF - Campbell *et al.* 2020 vs Controls	HF - Futter *et al.* 2011 vs Controls	HF - Rossing *et al.* 2016 vs Controls	HF - Campbell *et al.* 2020 vs HF - Futter *et al.* 2011	HF - Campbell *et al.* 2020 vs HF - Rossing *et al.* 2016	HF - Futter *et al.* 2011 vs HF - Rossing *et al.* 2016
e00005	109.82	187.43	85.23	32.81	3.35	5.71	2.60	3.96	330 (73)	159 (69)	57 (63)	220 (28)	**8.98E-73**	**7.51E-53**	**9.90E-43**	**1.27E-08**	7.85E-01	1.73E-01	**1.53E-02**
e01132	7567.23	9533.62	5334.46	4076.58	1.86	2.34	1.31	1.93	449 (100)	230 (100)	91 (100)	758 (98)	**3.25E-57**	**9.64E-41**	**7.27E-36**	**3.91E-03**	4.15E-01	**1.40E-03**	**2.45E-05**
e00340	1002.83	1225.14	631.76	483.00	2.08	2.54	1.31	2.12	444 (99)	218 (94)	88 (97)	616 (80)	**8.54E-56**	**7.42E-44**	**4.68E-30**	8.58E-02	1.00E+00	**6.59E-06**	**9.46E-06**
e08489	432.81	239.39	420.69	124.19	3.49	1.93	3.39	3.00	367 (82)	171 (74)	82 (90)	405 (52)	**3.58E-54**	**1.25E-49**	**4.19E-09**	**9.18E-15**	**1.01E-06**	1.00E+00	**3.97E-03**
e00421	915.62	1110.16	792.83	497.49	1.84	2.23	1.59	1.93	430 (96)	206 (89)	88 (97)	649 (84)	**1.09E-47**	**8.42E-37**	**4.58E-26**	**1.97E-06**	1.00E+00	5.46E-01	3.24E-01
e01461	1369.00	1411.32	1007.98	494.01	2.77	2.86	2.04	2.70	308 (69)	167 (72)	64 (70)	274 (35)	**1.11E-47**	**2.02E-35**	**1.13E-26**	**4.85E-08**	1.00E+00	1.00E+00	9.46E-01
e00152	83.46	113.23	57.82	30.39	2.75	3.73	1.90	2.94	275 (61)	144 (62)	60 (66)	202 (26)	**3.22E-47**	**2.89E-33**	**4.90E-28**	**1.49E-08**	9.68E-01	1.00E+00	9.52E-01
e16278	124.77	109.99	153.79	452.62	0.28	0.24	0.34	0.27	255 (57)	99 (43)	56 (62)	577 (75)	**1.70E-47**	**3.32E-31**	**1.42E-31**	**8.91E-07**	1.15E-01	1.00E+00	8.12E-02
e00274	39.68	68.75	28.18	11.30	3.51	6.09	2.50	4.15	259 (58)	127 (55)	40 (44)	165 (21)	**3.18E-47**	**5.88E-36**	**3.29E-27**	**2.04E-04**	1.00E+00	6.67E-02	**2.01E-02**
e05864	461.11	1209.12	679.37	2158.39	0.21	0.56	0.31	0.33	303 (67)	198 (86)	74 (81)	659 (85)	**5.63E-61**	**2.84E-60**	**8.57E-04**	**3.48E-09**	**6.88E-17**	6.73E-02	**7.08E-03**
e00134	27.12	29.90	19.38	7.98	3.40	3.75	2.43	3.38	249 (55)	107 (46)	42 (46)	152 (20)	**4.63E-41**	**1.01E-36**	**2.40E-16**	**2.18E-05**	6.74E-01	1.94E-01	1.00E+00
e15360	360.56	461.01	436.60	1229.80	0.29	0.37	0.36	0.32	420 (94)	210 (91)	82 (90)	711 (92)	**3.93E-41**	**1.42E-38**	**9.08E-12**	**2.07E-07**	**1.29E-02**	8.72E-01	1.00E+00
e03354	8333.34	8498.99	8961.50	5157.35	1.62	1.65	1.74	1.64	444 (99)	221 (96)	91 (100)	732 (95)	**1.68E-38**	**1.09E-27**	**7.12E-19**	**1.19E-11**	1.00E+00	1.00E+00	1.00E+00

e00957	50.91	56.22	43.98	18.93	2.69	2.97	2.32	2.72	257 (57)	83 (36)	50 (55)	150 (19)	**2.88E-41**	**8.14E-39**	**2.26E-08**	**9.82E-10**	**1.67E-04**	1.00E+00	1.88E-01
e05726	17.76	127.63	71.96	314.80	0.06	0.41	0.23	0.18	86 (19)	138 (60)	42 (46)	475 (61)	**5.05E-58**	**5.28E-58**	8.87E-02	**8.33E-05**	**5.32E-21**	**1.96E-04**	9.45E-02
e04294	85.67	79.88	57.83	26.01	3.29	3.07	2.22	3.09	195 (43)	100 (43)	38 (42)	102 (13)	**1.31E-34**	**6.53E-27**	**8.75E-18**	**4.67E-07**	1.00E+00	1.00E+00	1.00E+00
e00635	87.26	217.09	62.04	11.79	7.40	18.42	5.26	10.43	236 (53)	125 (54)	35 (38)	156 (20)	**5.92E-38**	**1.00E-25**	**9.16E-26**	6.96E-02	2.33E-01	**1.30E-02**	**1.60E-04**
e00131	43.55	112.11	50.08	20.20	2.16	5.55	2.48	3.21	213 (47)	131 (57)	47 (52)	174 (23)	**3.94E-36**	**4.35E-15**	**1.57E-30**	**4.04E-07**	**6.78E-06**	1.00E+00	1.59E-01
e01767	104.22	71.35	108.62	36.03	2.89	1.98	3.02	2.63	271 (60)	116 (50)	65 (71)	251 (32)	**1.04E-33**	**2.76E-28**	**1.47E-07**	**1.05E-12**	**1.44E-02**	1.00E+00	**7.76E-03**
e00259	211.34	280.19	132.13	142.17	1.49	1.97	0.93	1.57	427 (95)	215 (93)	81 (89)	534 (69)	**5.47E-38**	**3.28E-23**	**4.80E-27**	1.00E+00	**3.88E-02**	**5.10E-05**	**1.97E-08**

Abbreviations: **Avg. abundance** = average peptide abundance, **HF=** heart failure.
Peptides are ordered by increasing *p*-value in HF versus controls.
Peptides higher in disease are labeled in green, lower in red.
P-value<0.05 are bolded.

Table S4. Distribution of peptides in patients enrolled with acute (n=89) and chronic HF (n=682). Information is given for top 20 peptides providing the greatest discrimination between patients with HF (n=773) and controls (n=773). Average abundance, fold change and frequency in each analysed group are given. Peptide abundance between three groups was evaluated using Kruskal-Wallis test, followed by post-hoc analysis using Dunn test with Bonferroni method for *p*-value adjustment.

Peptide ID	Avg. abundance			Fold change			Frequency			*p*-value (Kruskal-Wallis)	p-value (post hoc test)		
	Acute HF	Chronic HF	Controls	Acute HF vs Controls	Chronic HF vs Controls	HF vs Controls	Acute HF, n (%)	Chronic HF, n (%)	Controls, n (%)		Acute HF vs Chronic HF	Acute HF vs Controls	Chronic HF vs Controls
e00005	85.49	136.00	32.81	2.61	4.14	3.96	61 (69)	485 (71)	220 (28)	**1.26E-72**	2.33E-01	**7.97E-11**	**7.88E-72**
e01132	7198.85	7983.42	4076.58	1.77	1.96	1.93	89 (100)	681 (100)	758 (98)	**1.02E-53**	1.00E+00	**1.19E-11**	**1.44E-51**
e00340	986.14	1030.79	483.00	2.04	2.13	2.12	88 (99)	662 (97)	616 (80)	**3.13E-51**	1.00E+00	**3.96E-13**	**5.06E-48**
e08489	501.79	356.68	124.19	4.04	2.87	3.00	78 (88)	542 (79)	405 (52)	**2.18E-50**	**3.30E-02**	**4.54E-19**	**6.34E-43**
e00421	835.19	975.62	497.49	1.68	1.96	1.93	87 (98)	637 (93)	649 (84)	**3.40E-48**	8.30E-01	**2.91E-08**	**1.63E-47**
e01461	1407.64	1330.12	494.01	2.85	2.69	2.70	50 (56)	489 (72)	274 (35)	**3.61E-49**	1.39E-01	**2.14E-06**	**2.62E-49**
e00152	62.22	92.89	30.39	2.05	3.06	2.94	50 (56)	429 (63)	202 (26)	**2.76E-48**	2.96E-01	**5.46E-07**	**3.61E-48**
e16278	98.34	127.08	452.62	0.22	0.28	0.27	53 (60)	357 (52)	577 (75)	**8.11E-47**	1.00E+00	**4.25E-11**	**1.56E-44**
e00274	39.85	47.97	11.30	3.53	4.25	4.15	49 (55)	377 (55)	165 (21)	**1.68E-46**	1.00E+00	**7.65E-09**	**1.89E-45**
e05864	389.50	752.94	2158.39	0.18	0.35	0.33	60 (67)	515 (76)	659 (85)	**1.54E-48**	**2.71E-03**	**4.60E-21**	**3.01E-39**
e00134	26.12	27.16	7.98	3.27	3.40	3.38	48 (54)	350 (51)	152 (20)	**7.50E-41**	1.00E+00	**5.48E-09**	**2.14E-39**
e15360	276.93	415.64	1229.79	0.23	0.34	0.32	87 (98)	625 (92)	711 (92)	**3.91E-41**	6.91E-02	**1.18E-15**	**3.31E-35**
e03354	7420.82	8592.35	5157.35	1.44	1.67	1.64	89 (100)	667 (98)	732 (95)	**2.48E-40**	8.35E-02	**1.24E-04**	**8.68E-41**
e00957	44.02	52.68	18.93	2.33	2.78	2.72	47 (53)	343 (50)	150 (19)	**1.81E-38**	1.00E+00	**7.85E-09**	**6.01E-37**
e05726	15.92	62.44	314.80	0.05	0.20	0.18	16 (18)	250 (37)	475 (61)	**1.76E-40**	**5.57E-03**	**5.88E-18**	**1.25E-32**
e04294	67.32	82.39	26.01	2.59	3.17	3.09	35 (39)	298 (44)	102 (13)	**1.03E-35**	1.00E+00	**1.68E-06**	**2.86E-35**
e00635	143.00	120.59	11.79	12.13	10.23	10.43	48 (54)	348 (51)	156 (20)	**3.89E-35**	1.00E+00	**4.97E-08**	**7.48E-34**
e00131	29.47	69.48	20.20	1.46	3.44	3.21	39 (44)	352 (52)	174 (23)	**1.97E-33**	**3.06E-02**	**4.59E-03**	**4.29E-34**

e01767	79.53	96.90	36.03	2.21	2.69	2.63	49 (55)	403 (59)	251 (32)	**5.27E-32**	8.15E-01	**2.51E-05**	**6.36E-32**
e00259	180.68	228.09	142.16	1.27	1.60	1.57	84 (94)	639 (94)	534 (69)	**4.44E-32**	2.35E-01	**3.73E-04**	**1.90E-32**

Abbreviations: **Avg**. **abundance** = average peptide abundance, **HF**= heart failure.
P-value<0.05 are bolded.
Peptides higher in disease are labeled in green, lower in red.

Table S5. Urinary peptide differences between HF subtypes. Significantly different peptides between HFrEF (n=117) and HFpEF (n=117) matched for sex, age, eGFR, systolic and diastolic blood pressure, diabetes and hypertension are listed, along with peptide characteristics (mass, retention time, sequence), frequency, average abundance and *p*-value (BH adjusted). Discriminatory metrics i.e. AUC, sensitivity, specificity, PPV and NPV are given. Significant peptides were defined as passing the criterion of $p<0.05$ and the frequency threshold of 30%. Peptides are sorted by increasing *p*-value.

ID	Mass [Da]	CE time [min]	Sequence	Protein name	HFrEF (n=117)		HFpEF (n=117)		*p*-value	AUC	Sensitivity	Specificity	PPV	NPV
					Freq. n (%)	Avg. abundance	Freq. n (%)	Avg. abundance						
e11211	2187.99	27.08	MGNYMDRVPTPQAIRAAQGL	Peptidoglycan recognition protein 1	91 (78)	365.34	106 (91)	714.24	2.11E-03	0.67	0.79	0.50	0.61	0.69
e12945	2423	33.98	DGpQGPpGSVGSVGGVGEKGEPGEAGN	Collagen alpha-1(XI) chain	24 (21)	25.54	52 (44)	84.60	7.83E-03	0.63	0.44	0.80	0.70	0.59
e11554	2234.92	32.98	GRTGDAGPVGPPGPpGppGpPGPPS	Collagen alpha-1(I) chain	29 (25)	43.56	56 (48)	174.38	8.52E-03	0.63	0.42	0.83	0.72	0.58
e11726	2258.96	26.79	pGVVGPQGAAGETGPmGERGHpGP	Collagen alpha-2(XI) chain	102 (87)	810.65	107 (91)	1490.21	8.57E-03	0.66	0.59	0.68	0.65	0.62
e06356	1600.7	40.07	PGVGERGppGpqGPPGP*	Collagen alpha-1(XVIII) chain	44 (38)	90.20	69 (59)	290.02	1.45E-02	0.64	0.29	0.94	0.85	0.57
e00307	876.39	35.1	GPpGpPGPpG	Collagen alpha-2(IX) chain	59 (50)	147.94	79 (68)	417.43	1.75E-02	0.64	0.34	0.91	0.80	0.58
e17438	3302.51	30.84	AGRpGEVGpPGpPGPAGEKGSPGADGPAGAPGTPGPQG	Collagen alpha-1(I) chain	23 (20)	43.19	48 (41)	125.05	2.29E-02	0.61	0.38	0.84	0.71	0.57
e06468	1612.76	23.41	ApGSKGDTGAKGEpGPVG	Collagen alpha-1(I) chain	43 (37)	41.86	19 (16)	10.93	2.54E-02	0.61	0.37	0.84	0.70	0.57
e09008	1885.87	21.25	GNAGPpGPpGPAGKEGGKGPR	Collagen alpha-1(I) chain	77 (66)	125.00	51 (44)	57.33	2.54E-02	0.63	0.56	0.67	0.63	0.60
e15611	2892.33	34.39	TGPQGpIGqpGpSGADGEPGPRGQQGLFGQ*	Collagen alpha-1(V) chain	60 (51)	197.48	81 (69)	503.18	2.63E-02	0.63	0.38	0.85	0.73	0.57
e17994	3443.54	25.67	PGPPGTSGHPGSPGSPGYQGPPGEpGqAGpSGpPGPPGA*	Collagen alpha-1(III) chain	73 (62)	204.24	88 (75)	404.51	3.23E-02	0.63	0.60	0.65	0.64	0.61
e10657	2101.91	22	AEGSpGRDGSpGAKGDRGETGp	Collagen alpha-1(I) chain	93 (79)	436.18	103 (88)	782.44	4.04E-02	0.63	0.71	0.52	0.60	0.64
e06511	1616.72	40.24	PGMFPSGTPGGPYGGAAP	Peflin	47 (40)	92.26	69 (59)	206.50	4.34E-02	0.62	0.55	0.68	0.64	0.60

Abbreviations: **AUC** = area under the receiver operating characteristic curve, **Avg**. **abundance** = average peptide abundance, **BH**= Benjamini-Hochberg, **Freq.**= frequency, **HF**= heart failure, **HFpEF**= HF with preserved ejection fraction, **HFrEF**= HF with reduced ejection fraction, **NPV** = negative predictive value, **PPV** = positive predictive value.

*Post-translational modification: deamidation (Q)

Table S6. List of the shortlisted *in silico* predicted proteases. Proteases that did not meet the criteria for shortlisting (i.e. at least one protease/CS association reported in the literature and the percentage of cleavage events above 1%) are marked in gray.

Protease Name	HF versus Controls				HFrEF versus Controls				HFmrEF versus Controls				HFpEF versus Controls			
	# Observed CS	# Predicted CS	#CS	% cleavage events	# Observed CS	# Predicted CS	#CS	% cleavage events	# Observed CS	# Predicted CS	#CS	% cleavage events	# Observed CS	# Predicted CS	#CS	% cleavage events
A disintegrin and metalloproteinase with thrombospondin motifs 4	2	308	310	2.31	2	254	256	2.28	1	113	114	2.31	0	122	122	2.37
Calpain-1 catalytic subunit	2	857	859	6.39	2	720	722	6.44	327	327	327	6.61	0	352	352	6.84
Calpain-2 catalytic subunit	2	856	858	6.38	2	709	711	6.34	0	324	324	6.55	0	354	354	6.88
Cathepsin B	2	583	585	4.35	2	483	485	4.33	2	226	228	4.61	0	215	215	4.18
Cathepsin K	21	382	403	3.00	16	314	330	2.94	8	151	159	3.22	8	147	155	3.01
Cathepsin L1	3	686	689	5.13	4	579	583	5.20	1	276	277	5.60	1	270	271	5.27
Cathepsin S	9	661	670	4.98	9	557	566	5.05	6	246	252	5.10	4	259	263	5.11
Meprin A subunit alpha	0	663	663	4.93	0	138	138	1.23	1	250	251	5.08	0	253	253	4.92
Interstitial collagenase	9	222	231	1.72	5	183	188	1.68	4	80	84	1.70	5	75	80	1.55
Macrophage metalloelastase	11	755	766	5.70	9	640	649	5.79	3	267	270	5.46	5	282	287	5.58
Collagenase 3	58	351	409	3.04	51	305	356	3.18	20	128	148	2.99	21	120	141	2.74
Matrix metalloproteinase-14	14	537	551	4.10	12	459	471	4.20	8	193	201	4.06	5	196	201	3.91
72 kDa type IV collagenase	42	338	380	2.83	33	295	328	2.93	18	121	139	2.81	13	116	129	2.51
Matrix metalloproteinase-25	24	383	407	3.03	18	336	354	3.16	10	125	135	2.73	8	165	173	3.36
Stromelysin-1	13	538	551	4.10	12	465	477	4.26	6	188	194	3.92	4	194	198	3.85
Matrilysin	2	854	856	6.37	2	719	721	6.43	2	322	324	6.55	1	337	338	6.57
Neutrophil collagenase	14	382	396	2.95	10	323	333	2.97	5	139	144	2.91	5	148	153	2.97
Matrix metalloproteinase-9	69	896	965	7.18	60	760	820	7.32	25	331	356	7.20	27	339	366	7.11
**Total # of retrieved CS**	**13441**				**11209**				**4945**				**5146**			

Abbreviations: **CS**= cleavage site, **HF**= heart failure, **HFmrEF**= HF with mid-range ejection fraction, **HFpEF**= HF with preserved ejection fraction, **HFrEF**= HF with reduced ejection fraction.

Figure S1. Workflow for matching patients with HF. Participants diagnosed with HFrEF (n=442), HFmrEF (n=144), HFpEF (n=187) were matched for sex, age, eGFR, systolic and diastolic blood pressure, diabetes and hypertension. This resulted in the selection of 117 individuals in each group.

***** When performing matching for patients with HFmrEF and HFpEF, HFmrEF patients that have been matched to HFrEF were considered. †When performing matching for patients with HFrEF and HFpEF, HFrEF patients that have been matched to HFmrEF were considered, similarly ‡HFpEF that have been matched to HFmrEF were considered.

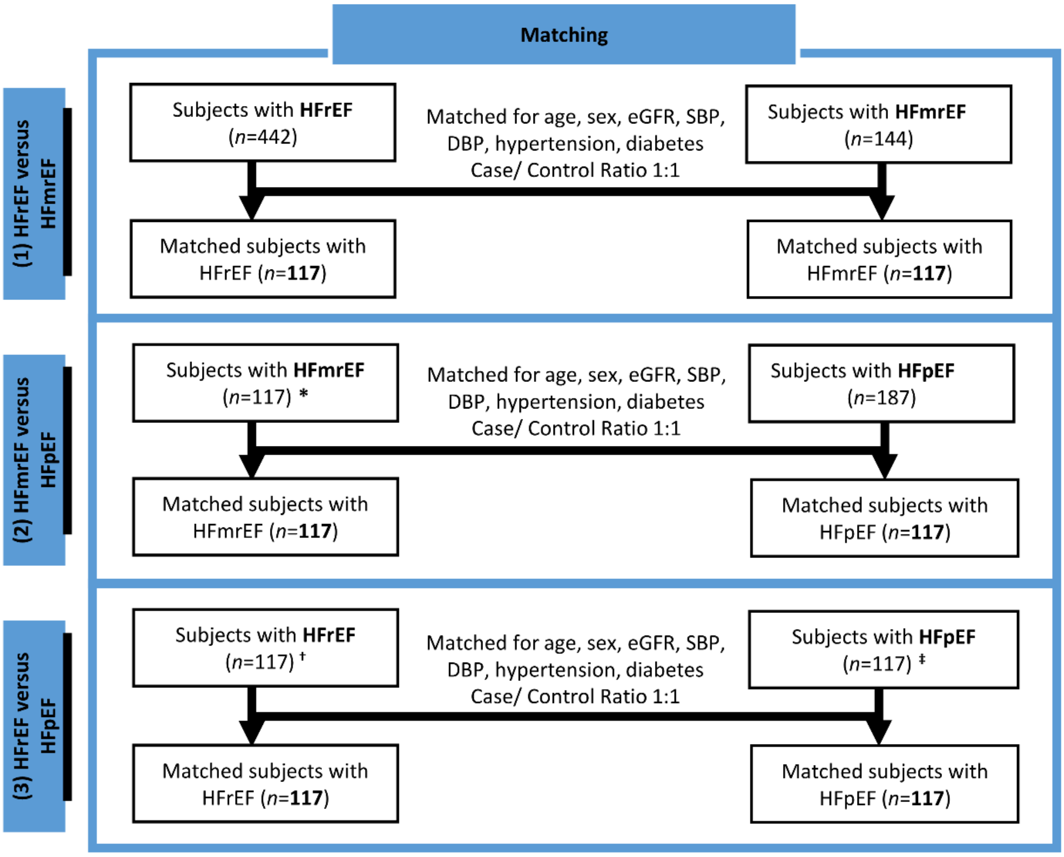

Figure S2. Analysis of HF patients stratified by cohort. Analysis was performed for three cohorts with the highest number of HF patients (i.e., Campbell *et al.* 2020 (n= 449), Futter *et al.* 2011 (n=231), and Rossing *et al.* 2016 (n=91)). Results are provided for peptides found to be significantly different between all patients with HF (n=773) and controls (n=773) including 577 peptides **(A and B)** and, separately, the 20 peptides with the greatest discrimination between HF and controls **(C)**. Correlation of **(A)** the peptide fold changes calculated in selected cohorts and in the complete HF cohort (n=773), in comparison to all controls included in the study and **(B)** average peptide abundance in three HF cohorts. **(C)** Box-plots displaying peptide abundance per cohort. Peptide abundance for the controls (n=773) is provided as a comparator. Mean is indicated with a red diamond.

A) Log$_2$ Fold change

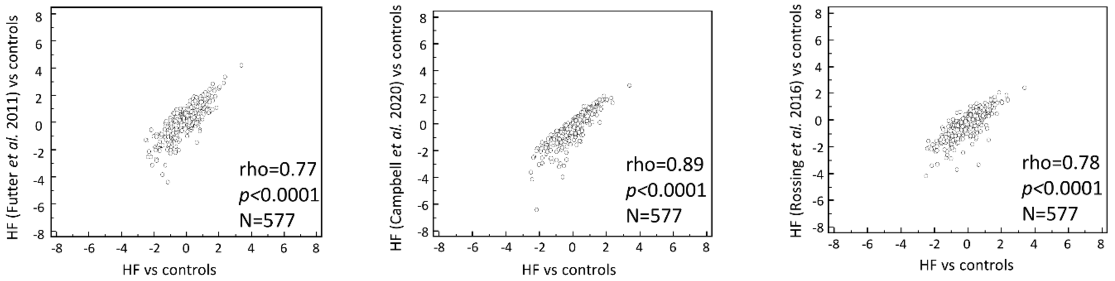

B) Average peptide intensity

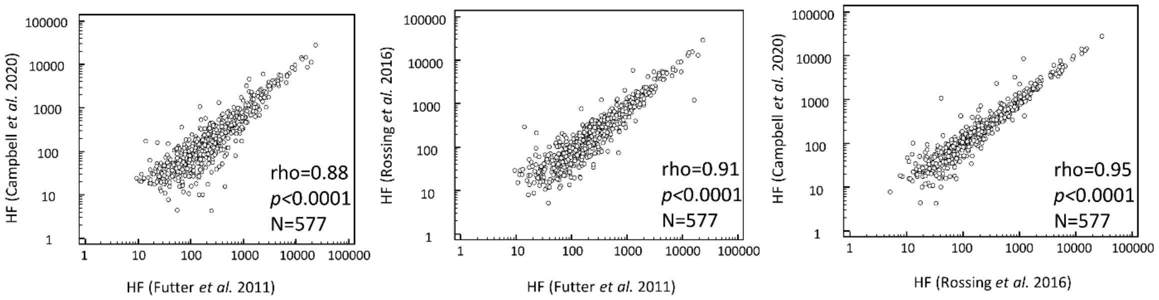

C)

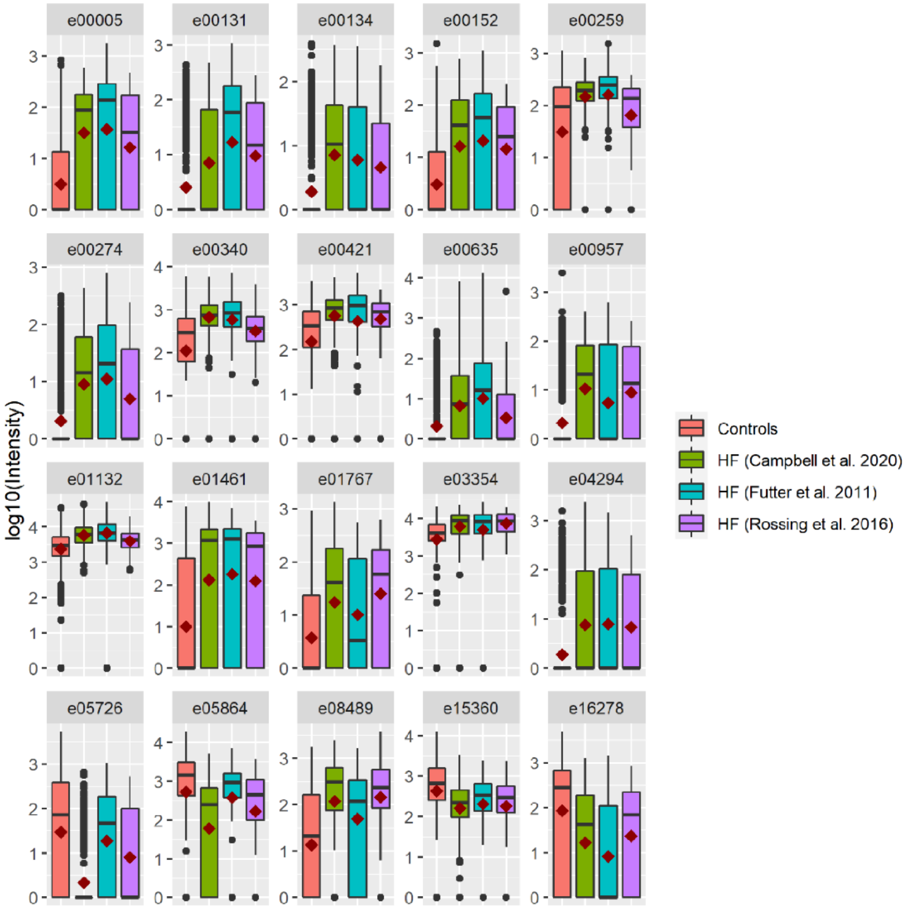

Figure S3. Analysis of HF patients stratified based on the enrolment status (acute and chronic HF). (A) Correlation of peptides significantly different between HF and controls when comparing average peptide abundance observed in patients with acute (n=89) and chronic HF (n=682). **(B)** Distribution of abundance for top 20 peptides exhibiting greatest discrimination between HF and controls (**Table 2**) in patients with acute and chronic HF and controls. Mean is indicated with a red diamond.

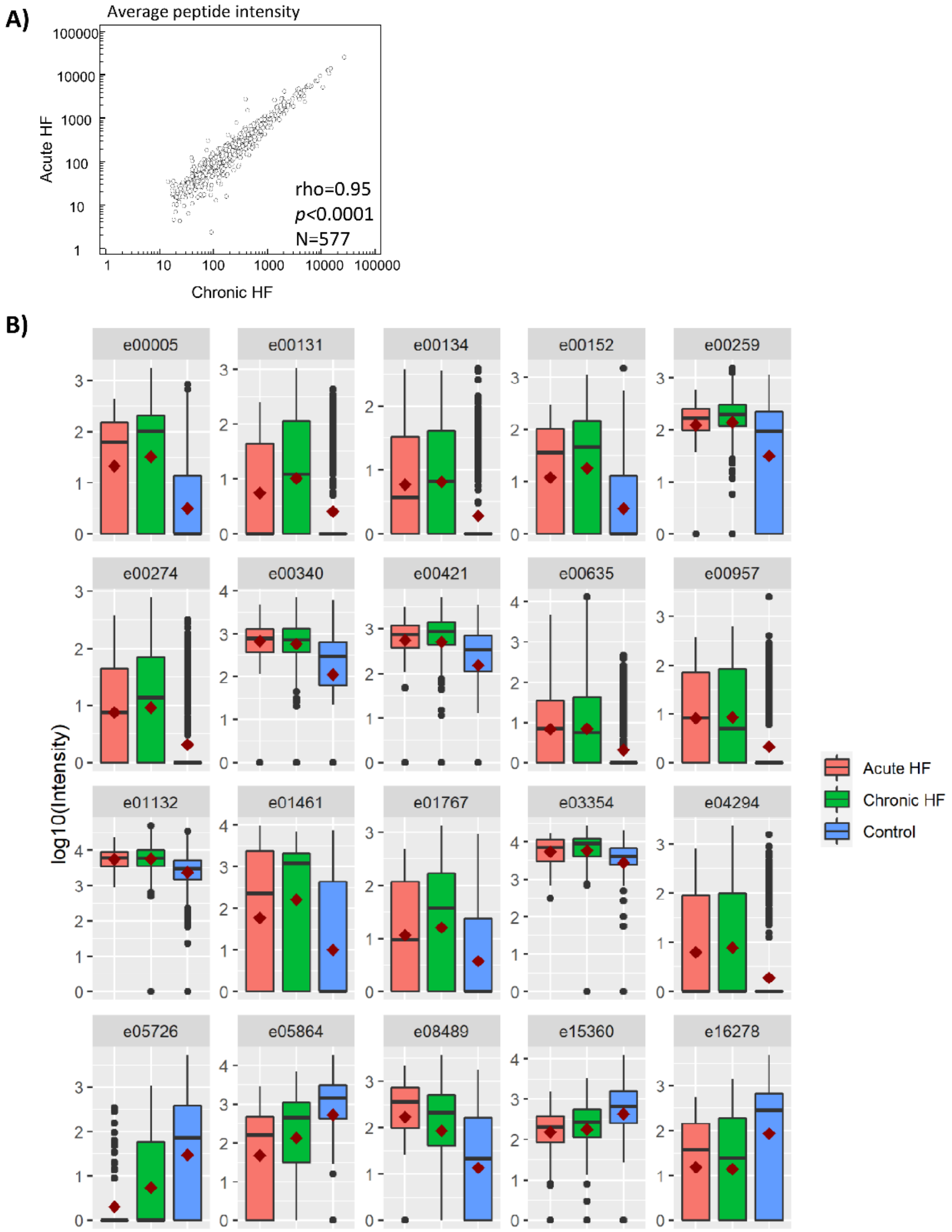

Figure S4. Comparison of CKD and HF associated urinary peptides. **(A)** The comparison was performed between 273 CKD associated peptides defined previously when comparing patients with CKD and normal controls (Good *et al.*, 2010) and 577 HF associated peptides defined in this study. 71 peptides were found overlapping between these two sets of biomarkers. **(B)** Correlation analysis of fold changes for 71 common peptides is presented.

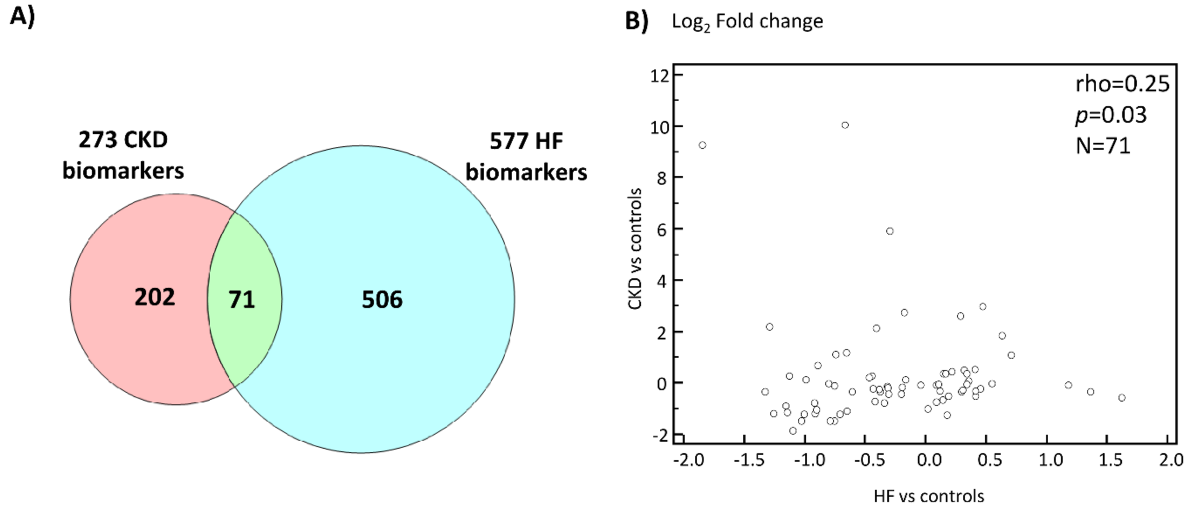

Figure S5. Summary of the consensus clustering results. Segregation of patients with HF only into clusters for k = 2-8 solutions. Percentage of patients assigned to the class is given. Chi-squared test was applied to assess differences in the distribution of patients within the class.

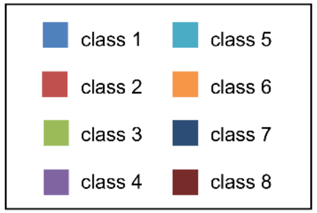

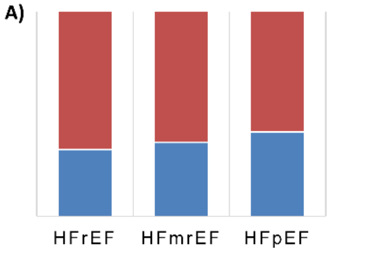

K=2	HFrEF [%]	HFmrEF [%]	HFpEF [%]	*p*-value
class 1	33	36	41	*p*=0.6405
class 2	67	64	59	*p*=0.7727

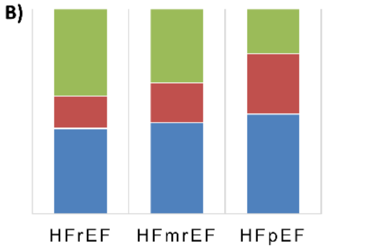

K=3	HFrEF [%]	HFmrEF [%]	HFpEF [%]	*p*-value
class 1	29	31	37	*p*=0.5850
class 2	12	15	25	*p*=0.0690
class 3	17	17	17	*p*=1.0000
class 4	42	37	21	*p*=0.0271

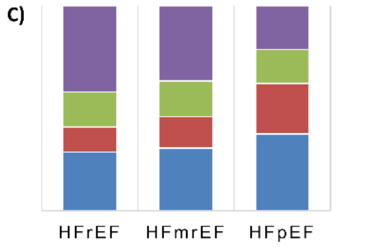

K=4	HFrEF [%]	HFmrEF [%]	HFpEF [%]	*p*-value
class 1	19	23	25	*p*=0.6584
class 2	14	17	28	*p*=0.0631
class 3	13	14	11	*p*=0.8318
class 4	29	25	17	*p*=0.2065
class 5	25	22	19	*p*=0.6643

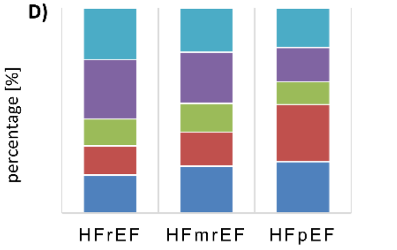

K=5	HFrEF [%]	HFmrEF [%]	HFpEF [%]	*p*-value
class 1	19	23	25	*p*=0.6584
class 2	14	17	28	*p*=0.0631
class 3	13	14	11	*p*=0.8318
class 4	29	25	17	*p*=0.2065
class 5	25	22	19	*p*=0.6643

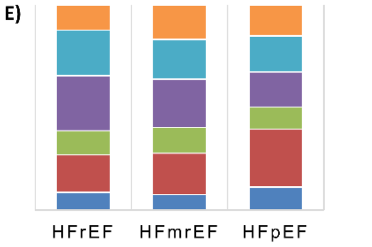

K=6	HFrEF [%]	HFmrEF [%]	HFpEF [%]	*p*-value
class 1	9	8	11	*p*=0.7788
class 2	18	20	28	*p*=0.2801
class 3	12	13	11	*p*=0.9200
class 4	27	24	17	*p*=0.3129
class 5	22	19	18	*p*=0.8022
class 6	12	17	15	*p*=0.6493

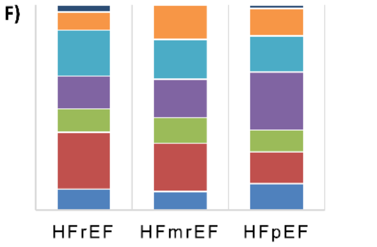

K=7	HFrEF [%]	HFmrEF [%]	HFpEF [%]	*p*-value
class 1	10	9	13	*p*=0.6661
class 2	28	24	16	*p*=0.1926
class 3	11	13	11	*p*=0.8920
class 4	16	19	28	*p*=0.1561
class 5	22	19	18	*p*=0.8022
class 6	9	17	13	*p*=0.2921
class 7	3	0	2	*p*=1.0000

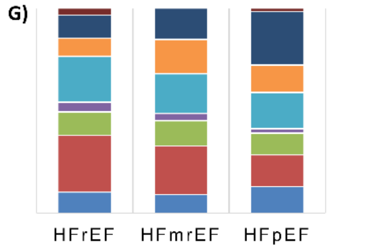

K=8	HFrEF [%]	HFmrEF [%]	HFpEF [%]	*p*-value
class 1	10	9	13	*p*=0.6661
class 2	28	24	16	*p*=0.1926
class 3	11	13	11	*p*=0.8920
class 4	5	3	2	*p*=0.4966
class 5	22	19	18	*p*=0.8022
class 6	9	17	13	*p*=0.2921
class 7	11	15	26	*p*=0.0308
class 8	3	0	2	*p*=1.0000

Figure S6. Performance of biomarker panels discriminating between HF subtypes. The urinary peptidomics data from patients with HFrEF (n=117), HFmrEF (n=117) and HFpEF (n=117) matched for sex, age, eGFR, SBP, DBP, diabetes and hypertension, were randomly divided into two sets (training set, n=70 and test set, n=47). Thirty peptides with the highest AUC were selected in the training set (in each pairwise analysis separately) and combined using SVM, followed by optimisation of SVM parameters. Performance of biomarkers was assessed in the test set. Receiving operating characteristics analysis based on test set data was conducted for combination of biomarkers discriminating **(A)** patients with HFrEF (n=47) from patients with HFmrEF (n=47), **(B)** patients with HFmrEF (n=47) from patients with HFpEF (n=47) and **(C)** patients with HFrEF (n=47) from patients with HFpEF (n=47). Information on specificity and sensitivity of the model at the pre-specified cut-off (based on the Youden index J) is provided.

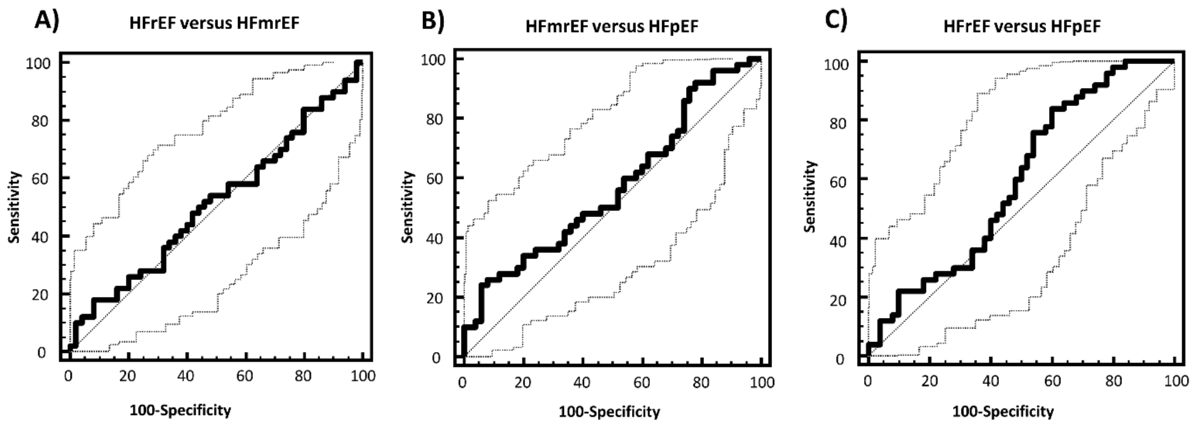

Test set	Performance
Sample size (n)	94
HFrEF / HFmrEF (n)	47/ 47
Area under the curve	0.515
95% Confidence interval	0.413 to 0.616
Sensitivity/ Specificity	18% / 92%
p-value	*p*=0.8002

Test set	Performance
Sample size (n)	94
HFmrEF / HFpEF (n)	47/ 47
Area under the curve	0.571
95% Confidence interval	0.468 to 0.670
Sensitivity/ Specificity	26% / 92%
p-value	*p*=0.2179

Test set	Performance
Sample size (n)	94
HFrEF / HFpEF (n)	47/ 47
Area under the curve	0.596
95% Confidence interval	0.493 to 0.693
Sensitivity/ Specificity	84% / 40%
p-value	*p*=0.0971

Figure S7. Urinary peptides differences between HF subtypes and controls. Volcano plot showing distribution of the identified sequenced peptides between matched controls and patients with HFrEF, HFmrEF and HFpEF. Directionality of the difference, magnitude as well as significance level (BH adjusted *p*-value) are displayed. Discrimination between collagen and non-collagen derived peptides is provided. Peptides originated from proteins for which at least 10 significant peptides were identified ($p<0.05$, BH adjusted) when comparing all patients with HF and controls are color-coded. Peptides with $p<0.05$ (BH adjusted) are marked in grey. **Abbreviations**: **COL1A1**= Collagen alpha-1(I) chain; **COL1A2**= Collagen alpha-2(I) chain; **COL2A1**= Collagen alpha-1(II) chain; **COL3A1**= Collagen alpha-1(III) chain; **FGA**= Fibrinogen alpha chain; **UMOD**= Uromodulin.

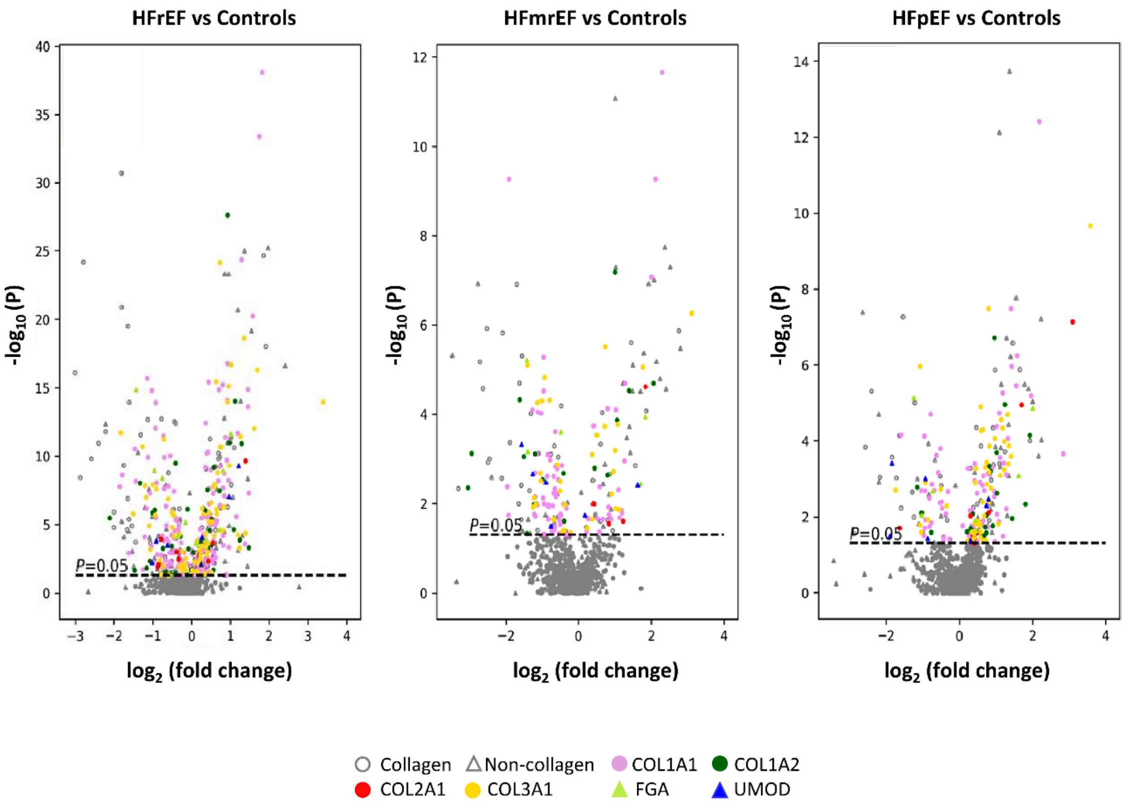

Supplementary methods. Detailed information on CE-MS analysis, peptide sequencing as well as bioinformatics are provided.

CE-MS

Preparation and measurements of urine samples using CE-MS were conducted as described previously (1). Briefly, 0.7 mL urine was diluted with 0.7 mL of an aqueous solution of 2 M urea, 10 mM NH_4OH, and 0.02 % sodium dodecyl sulfate. To remove proteins of higher molecular mass, the sample was filtered using Centrisart ultracentrifugation filter devices (20 kDa molecular weight cut-off; Sartorius, Germany), followed by desalting using a PD-10 desalting column (GE Healthcare, Germany) and lyophilisation. Lyophilized samples were resuspended in HPLC-grade water shortly before analysis. CE-MS analysis was performed with a P/ACE MDQ CE (Beckman Coulter, USA) coupled to a micro-TOF-MS (Bruker Daltonic, Germany). RAW MS data were evaluated using MosaFinder (2) applying a probabilistic clustering algorithm and using both isotopic distributions and conjugated masses for charge state determination. Peptide mass, migration time, and signal intensity were calibrated using internal urinary standard peptides to assure the comparability between different datasets. Specifically, TOF-MS data were calibrated utilizing a list of reference masses applying linear regression. CE-migration time was calibrated using a locally weighted regression algorithm with internal standard peptides as reference. The signal intensity was normalised using a linear regression based on 29 collagen fragments that are generally not affected by disease and serve as internal standards, described in detail in (3). This enabled correction for variability of migration time and intensity. Missing values were interpreted as zero.

Sequencing of peptides

Identified HF biomarkers were assigned *in silico* to sequenced peptides from the Human Urinary Proteome database as described elsewhere (2). Briefly, urinary peptides were

fragmented using Orbitrap MS coupled to CE or liquid chromatography (4). Fragmentation spectra were matched to the protein sequences from up-to-date databases (International Protein Index (5), Reference sequence database at NCBI (6) and UniProt Knowledgebase (7)) using Proteome Discoverer 1.4 (Thermo Scientific, Bremen, Germany). The following search parameters were applied: 1) precursor mass tolerance: 5 ppm, 2) fragment mass tolerance: 50 mDa and 3) variable post-translational modifications: hydroxylation of lysine and proline, and oxidation of methionine.

Bioinformatic analysis

Proteases responsible for the generation of the urinary peptide fragments were predicted using Proteasix (8). Protease/cleavage site associations were retrieved based on matching against cleavage site associations from the literature as well as the probability of cleavage by a protease based on MEROPS specificity matrices. Predicted proteases as well as proteins corresponding to sequenced urinary peptides were included as an input for functional analysis. Functional analysis was performed with the Metascape (9), with Reactome Gene Sets used as an ontology source. All genes in the genome were used as the enrichment background. Terms with a *p*-value < 0.01, a minimum count of 3, and an enrichment factor > 2.5 were collected and grouped into clusters based on their membership similarities. *P*-values were calculated based on the accumulative hypergeometric distribution, followed by Benjamini-Hochberg adjustments. Kappa scores were used as the similarity metric when performing hierarchical clustering on the enriched terms, and sub-trees with a similarity of >0.3 were considered a cluster. The most statistically significant term within a cluster was chosen to represent the cluster. To capture the relationship between the terms, a subset of enriched terms was selected and rendered as a network plot, where terms with a similarity >0.3 are connected by edges. The network was visualized using Cytoscape (v3.5.0) (10).

References

1. Mischak H, Vlahou A, Ioannidis JP. Technical aspects and inter-laboratory variability in native peptide profiling: the CE-MS experience. Clin Biochem 2013;**46**:432-43.
2. Latosinska A, Siwy J, Mischak H, Frantzi M. Peptidomics and proteomics based on CE-MS as a robust tool in clinical application: The past, the present, and the future. Electrophoresis 2019;**40**:2294-308.
3. Jantos-Siwy J, Schiffer E, Brand K, Schumann G, Rossing K, Delles C, Mischak H, Metzger J. Quantitative urinary proteome analysis for biomarker evaluation in chronic kidney disease. J Proteome Res 2009;**8**:268-81.
4. Klein J, Papadopoulos T, Mischak H, Mullen W. Comparison of CE-MS/MS and LC-MS/MS sequencing demonstrates significant complementarity in natural peptide identification in human urine. Electrophoresis 2014;**35**:1060-4.
5. Kersey PJ, Duarte J, Williams A, Karavidopoulou Y, Birney E, Apweiler R. The International Protein Index: an integrated database for proteomics experiments. Proteomics 2004;**4**:1985-8.
6. O'Leary NA, Wright MW, Brister JR, Ciufo S, Haddad D, McVeigh R, Rajput B, Robbertse B, Smith-White B, Ako-Adjei D, Astashyn A, Badretdin A, Bao Y, Blinkova O, Brover V, Chetvernin V, Choi J, Cox E, Ermolaeva O, Farrell CM, Goldfarb T, Gupta T, Haft D, Hatcher E, Hlavina W, Joardar VS, Kodali VK, Li W, Maglott D, Masterson P, McGarvey KM, Murphy MR, O'Neill K, Pujar S, Rangwala SH, Rausch D, Riddick LD, Schoch C, Shkeda A, Storz SS, Sun H, Thibaud-Nissen F, Tolstoy I, Tully RE, Vatsan AR, Wallin C, Webb D, Wu W, Landrum MJ, Kimchi A, Tatusova T, DiCuccio M, Kitts P, Murphy TD, Pruitt KD. Reference sequence (RefSeq) database at NCBI: current status, taxonomic expansion, and functional annotation. Nucleic Acids Res 2016;**44**:D733-45.

7. UniProt C. UniProt: a worldwide hub of protein knowledge. Nucleic Acids Res 2019;**47**:D506-D15.
8. Klein J, Eales J, Zurbig P, Vlahou A, Mischak H, Stevens R. Proteasix: a tool for automated and large-scale prediction of proteases involved in naturally occurring peptide generation. Proteomics 2013;**13**:1077-82.
9. Zhou Y, Zhou B, Pache L, Chang M, Khodabakhshi AH, Tanaseichuk O, Benner C, Chanda SK. Metascape provides a biologist-oriented resource for the analysis of systems-level datasets. Nat Commun 2019;**10**:1523.
10. Shannon P, Markiel A, Ozier O, Baliga NS, Wang JT, Ramage D, Amin N, Schwikowski B, Ideker T. Cytoscape: a software environment for integrated models of biomolecular interaction networks. Genome Res 2003;**13**:2498-504.
