# Supplementary figures and images for: Urinary peptides in heart failure: a link to molecular pathophysiology
Source: Eur J Heart Fail. 2021 May 7;23(11):1875–87. doi: 10.1002/ejhf.2195 (PMC9291452; doi:10.1002/ejhf.2195)

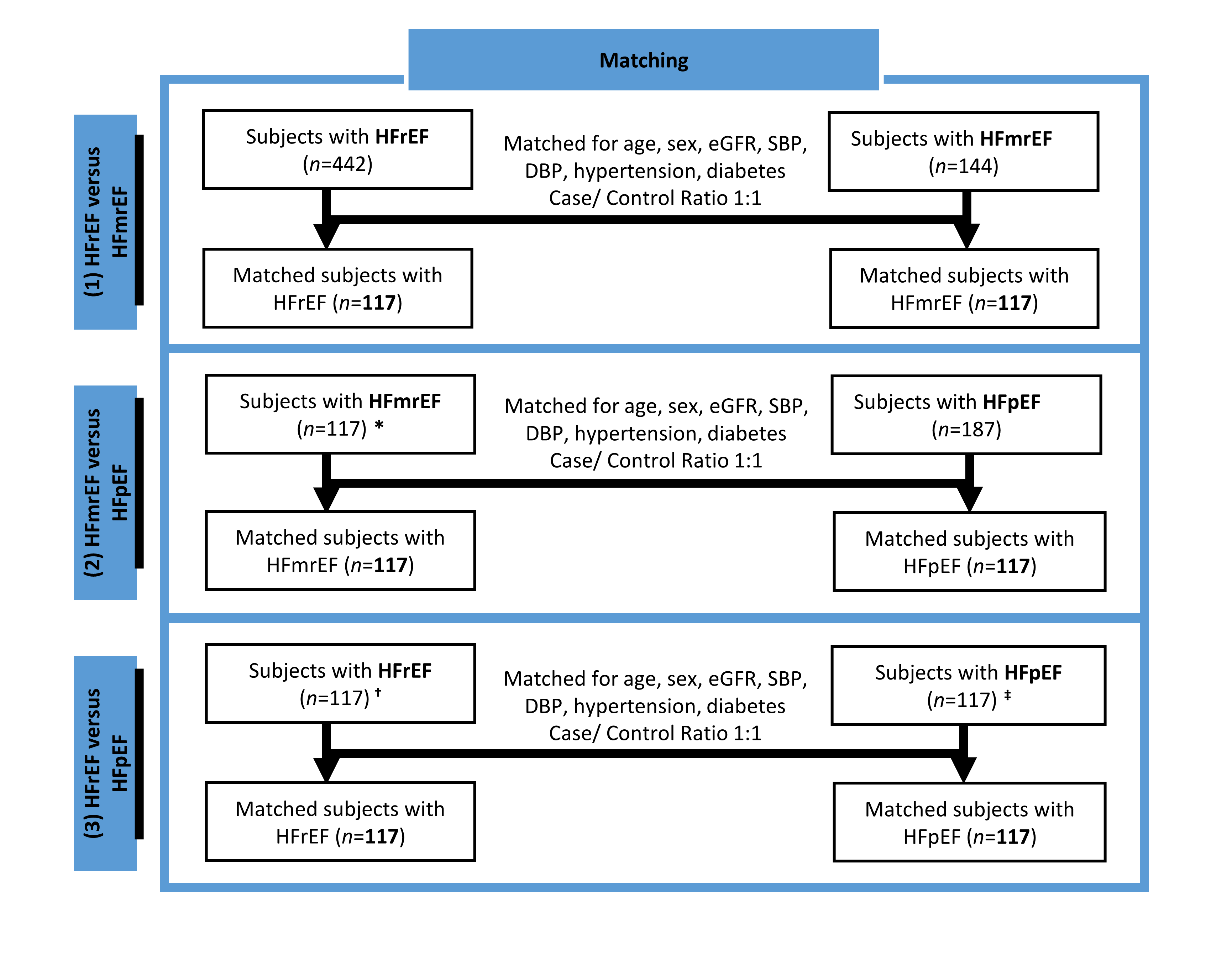

Supplement: Supplementary file 2 — Figure S1. Workflow for matching patients with heart failure. Participants diagnosed with HFrEF (n = 442), HFmrEF (n = 144), HFpEF (n = 187) were matched for sex, age, eGFR, systolic and diastolic blood pressure, diabetes and hypertension. This resulted in the selection of 117 individuals in each group. * When performing matching for patients with HFmrEF and HFpEF, HFmrEF patients that have been matched to HFrEF were considered. † When performing matching for patients with HFrEF and HFpEF, HFrEF patients that have been matched to HFmrEF were considered, similarly ‡ HFpEF that have been matched to HFmrEF were considered. [file EJHF-23-1875-s006.tif]

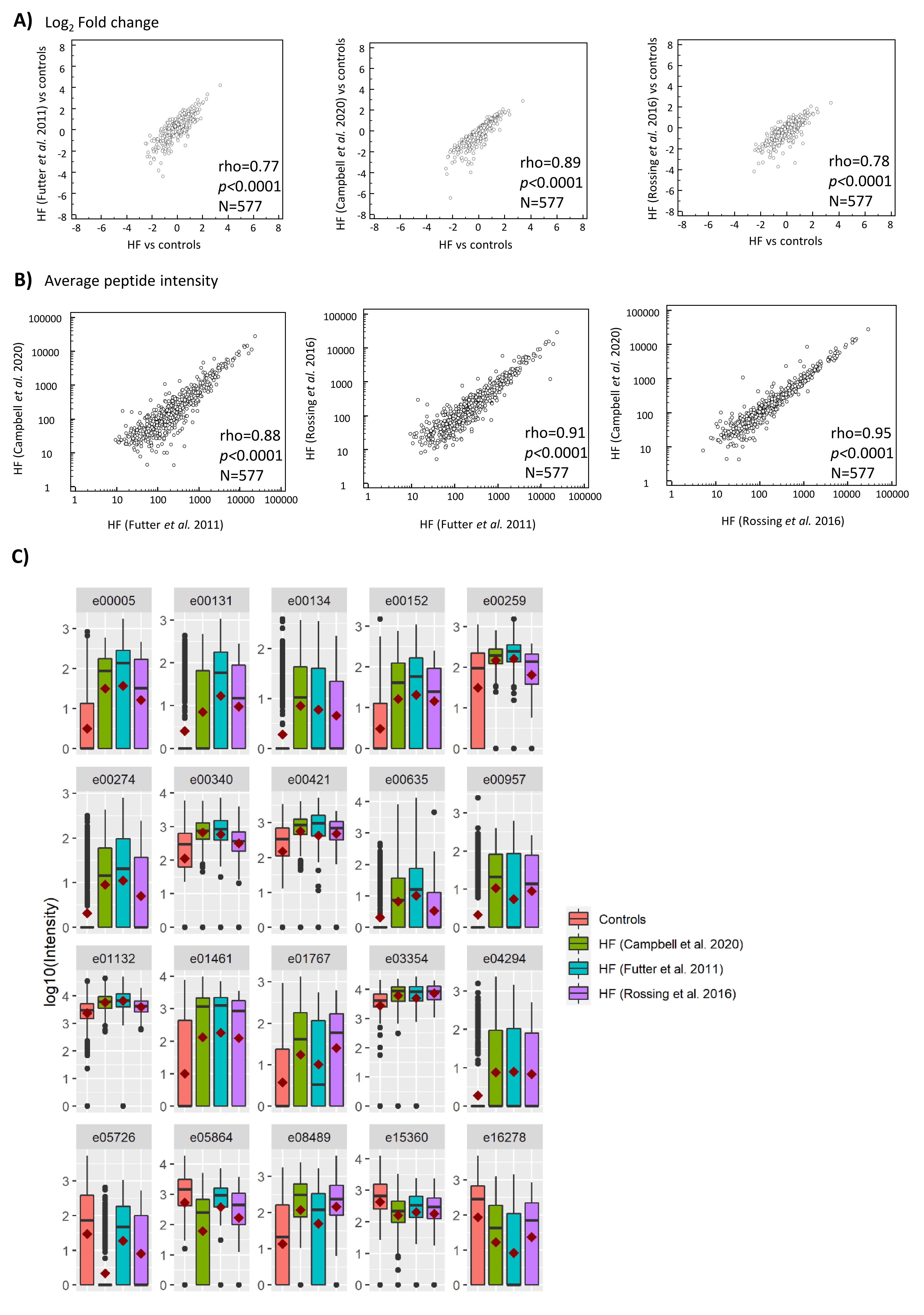

Supplement: Supplementary file 3 — Figure S2. Analysis of heart failure patients stratified by cohort. Analysis was performed for three cohorts with the highest number of HF patients [i.e. Campbell et al. 2020 (n = 449), Futter et al. 2011 (n = 231), and Rossing et al. 2016 (n = 91)]. Results are provided for peptides found to be significantly different between all patients with HF (n = 773) and controls (n = 773) including 577 peptides (A and B) and, separately, the 20 peptides with the greatest discrimination between HF and controls (C). Correlation of (A) the peptide fold changes calculated in selected cohorts and in the complete HF cohort (n = 773), in comparison to all controls included in the study and (B) average peptide abundance in three HF cohorts. (C) Box‐plots displaying peptide abundance per cohort. Peptide abundance for the controls (n = 773) is provided as a comparator. Mean is indicated with a red diamond. [file EJHF-23-1875-s008.tif]

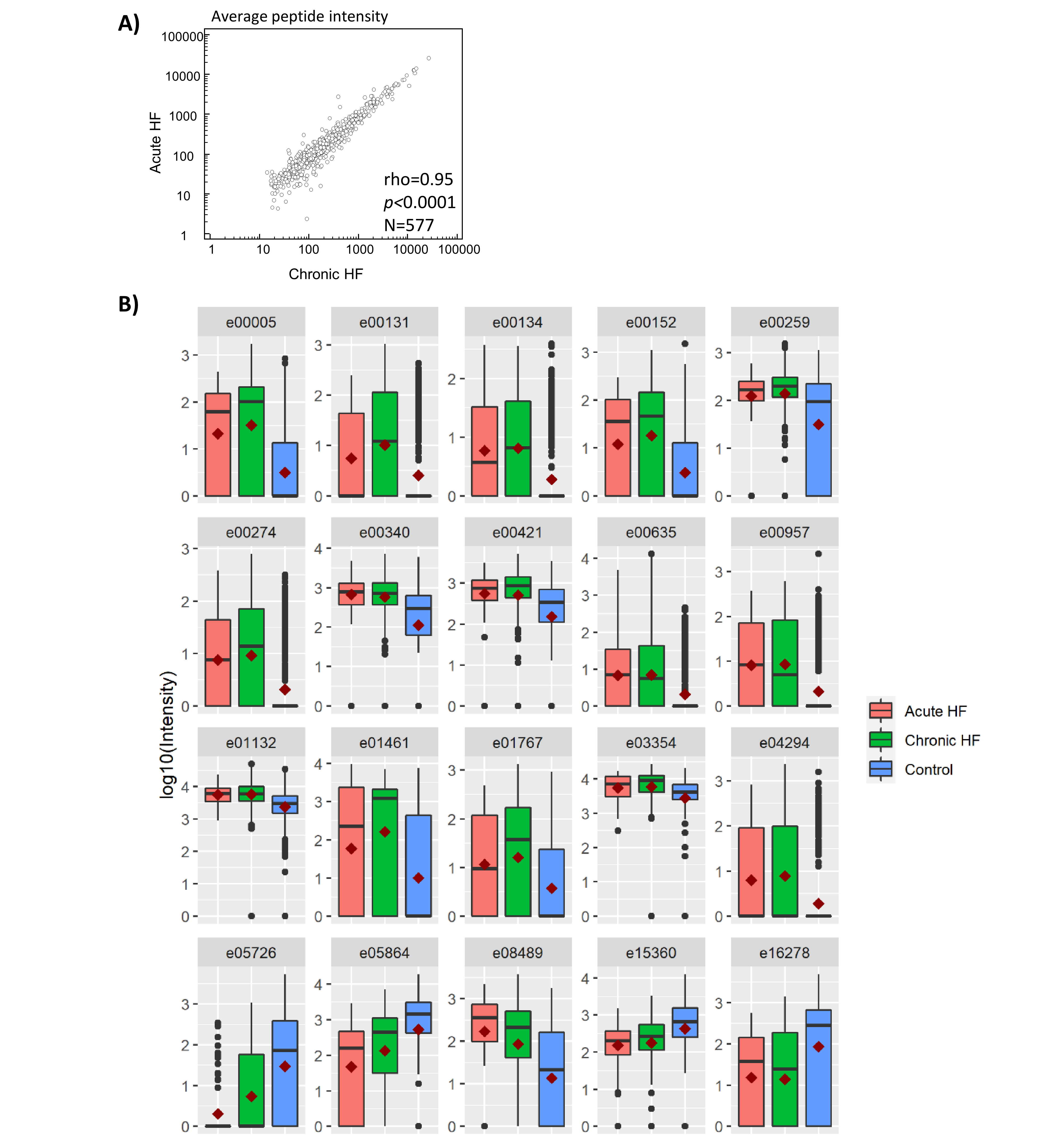

Supplement: Supplementary file 4 — Figure S3. Analysis of heart failure patients stratified based on the enrolment status (acute and chronic heart failure). (A) Correlation of peptides significantly different between HF and controls when comparing average peptide abundance observed in patients with acute (n = 89) and chronic HF (n = 682). (B) Distribution of abundance for top 20 peptides exhibiting greatest discrimination between HF and controls (Table 2 ) in patients with acute and chronic HF and controls. Mean is indicated with a red diamond. [file EJHF-23-1875-s004.tif]

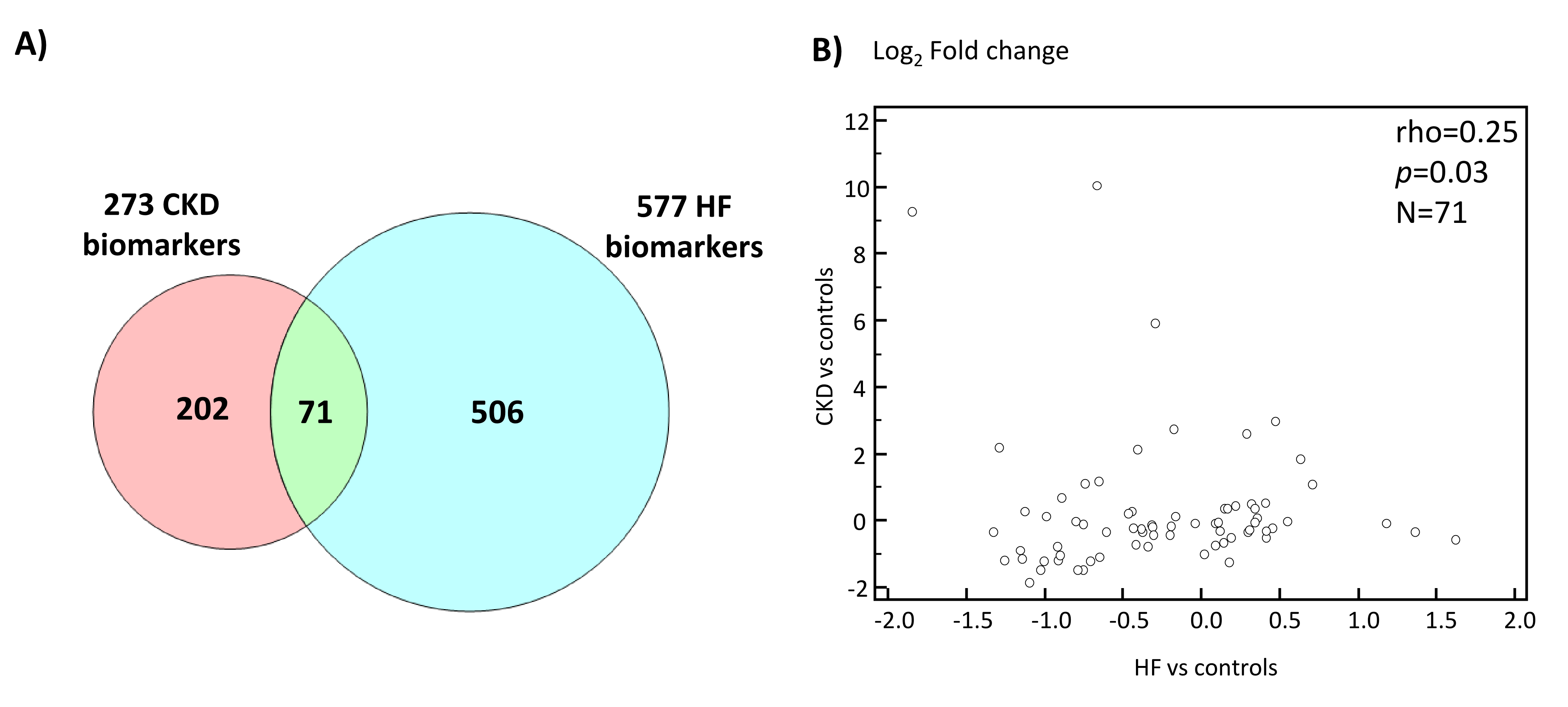

Supplement: Supplementary file 5 — Figure S4. Comparison of chronic kidney disease and heart failure associated urinary peptides. (A) The comparison was performed between 273 CKD associated peptides defined previously when comparing patients with CKD and normal controls (Good et al., 2010) and 577 HF associated peptides defined in this study. Seventy‐one peptides were found overlapping between these two sets of biomarkers. (B) Correlation analysis of fold changes for 71 common peptides is presented. [file EJHF-23-1875-s003.tif]

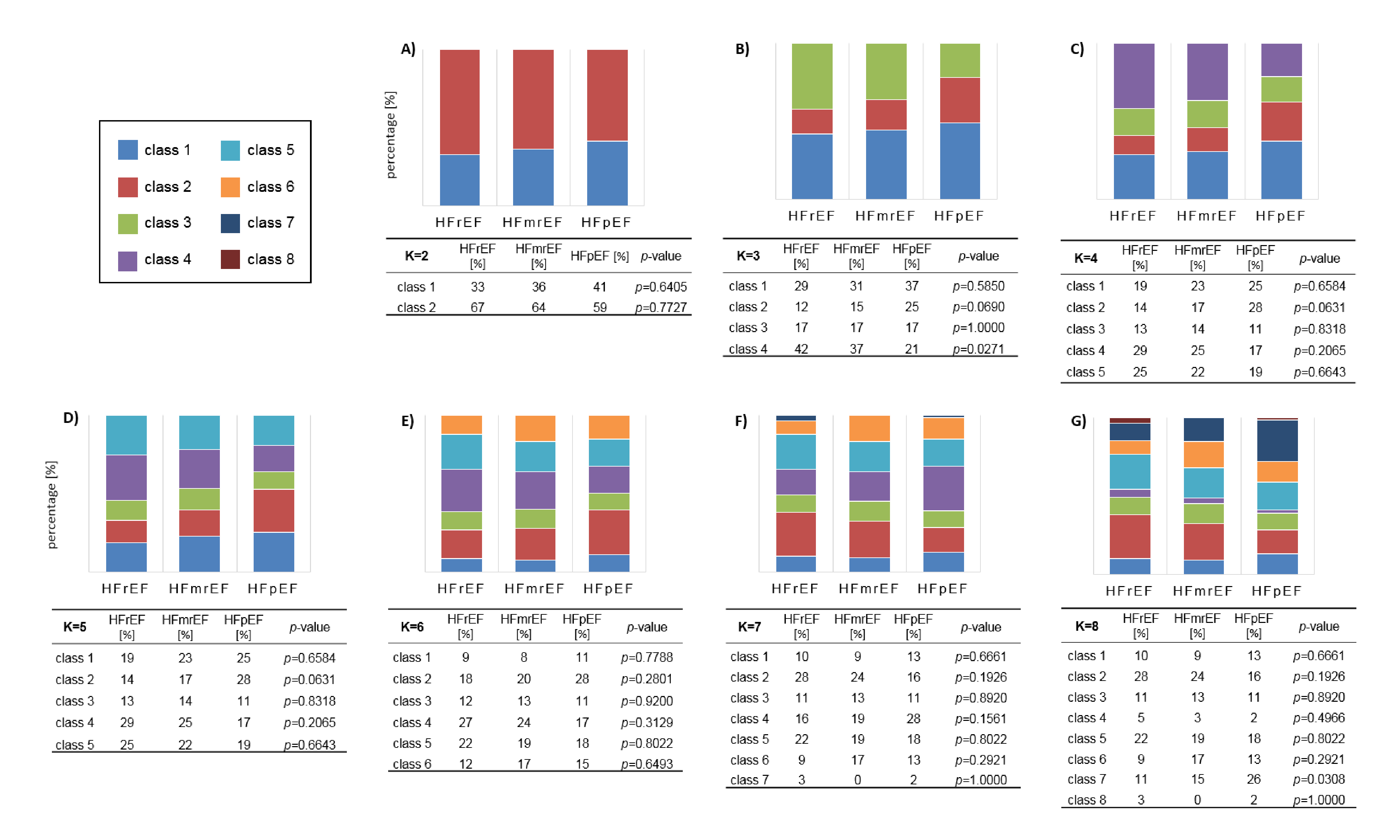

Supplement: Supplementary file 6 — Figure S5. Summary of the consensus clustering results. Segregation of patients with HF only into clusters for k = 2–8 solutions. Percentage of patients assigned to the class is given. Chi‐squared test was applied to assess differences in the distribution of patients within the class. [file EJHF-23-1875-s005.tif]

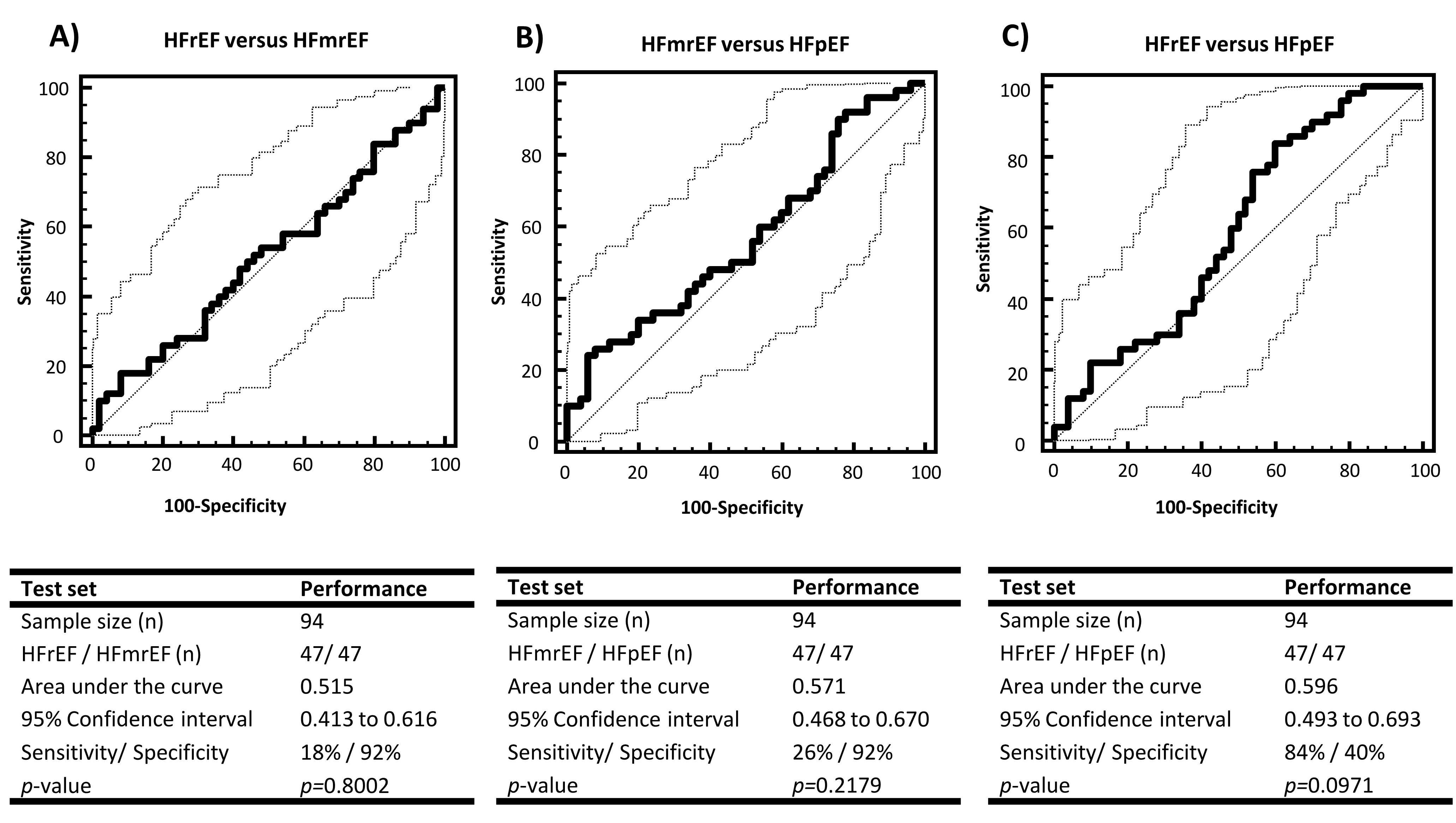

Supplement: Supplementary file 7 — Figure S6. Performance of biomarker panels discriminating between heart failure subtypes. The urinary peptidomics data from patients with HFrEF (n = 117), HFmrEF (n = 117) and HFpEF (n = 117) matched for sex, age, eGFR, SBP, DBP, diabetes and hypertension, were randomly divided into two sets (training set, n = 70 and test set, n = 47). Thirty peptides with the highest AUC were selected in the training set (in each pairwise analysis separately) and combined using SVM, followed by optimisation of SVM parameters. Performance of biomarkers was assessed in the test set. Receiving operating characteristic analysis based on test set data was conducted for combination of biomarkers discriminating (A) patients with HFrEF (n = 47) from patients with HFmrEF (n = 47), (B) patients with HFmrEF (n = 47) from patients with HFpEF (n = 47) and (C) patients with HFrEF (n = 47) from patients with HFpEF (n = 47). Information on specificity and sensitivity of the model at the pre‐specified cut‐off (based on the Youden index J) is provided. [file EJHF-23-1875-s001.tif]

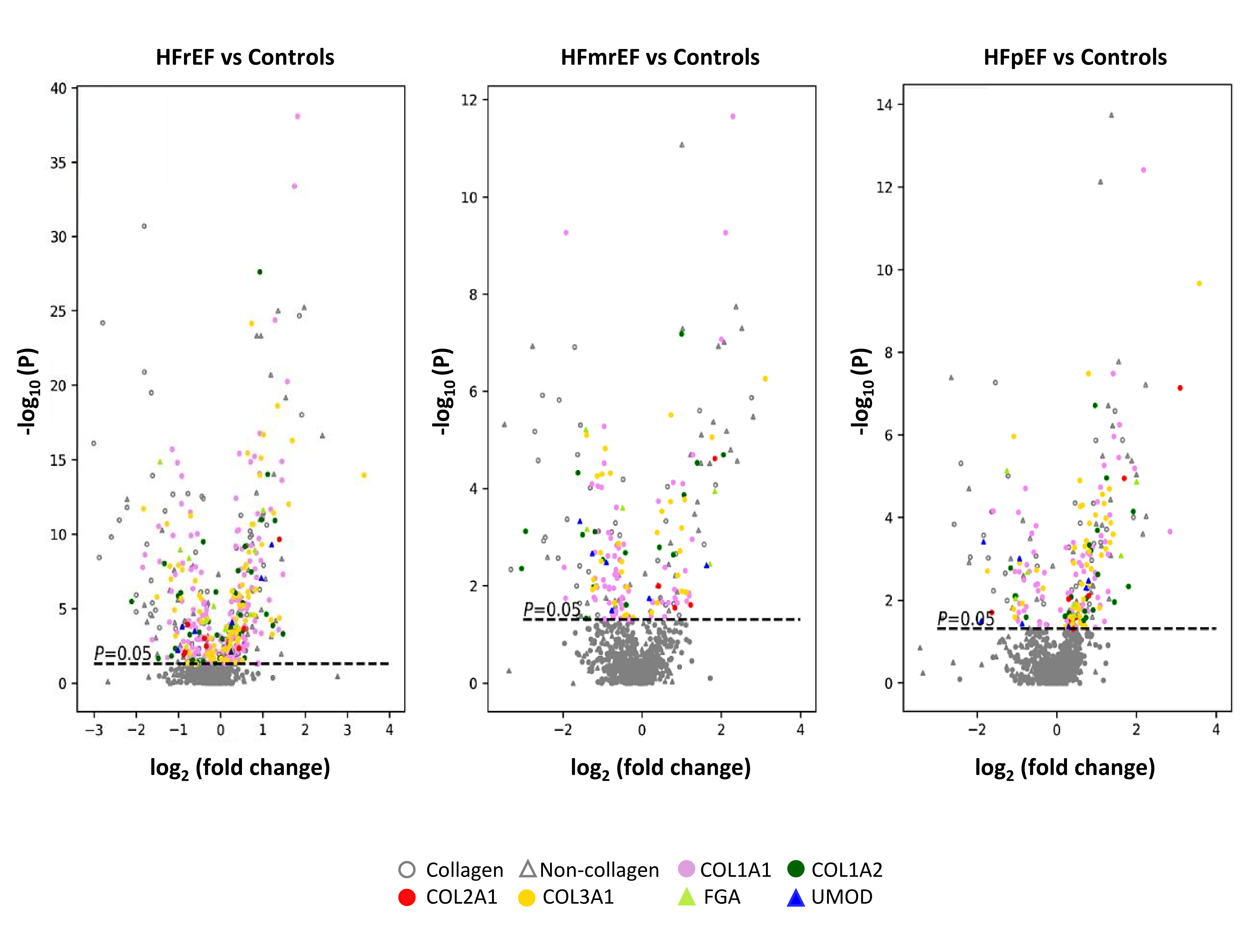

Supplement: Supplementary file 8 — Figure S7. Urinary peptides differences between heart failure subtypes and controls. Volcano plot showing distribution of the identified sequenced peptides between matched controls and patients with HFrEF, HFmrEF and HFpEF. Directionality of the difference, magnitude as well as significance level (BH adjusted P‐value) are displayed. Discrimination between collagen and non‐collagen derived peptides is provided. Peptides originated from proteins for which at least 10 significant peptides were identified (P < 0.05, BH adjusted) when comparing all patients with HF and controls are color‐coded. Peptides with P < 0.05 (BH adjusted) are marked in grey. [file EJHF-23-1875-s007.tif]
